# Probiotics use environmentally friendly calcium lignosulfonate as an energy source to MICP-acting on concrete and soil remediation

**DOI:** 10.3389/fmicb.2026.1826075

**Published:** 2026-05-11

**Authors:** Zi-jun Yan, Fang-yu Song, Wen-hao Xu, Ke-qin Fan, Peng-yu Zhao, Mei Tong, Chong Yu, Siqing Kong, Tong Chen, Xian-wen Ye, Liang-jing Xia

**Affiliations:** 1 Yunnan Key Laboratory of Pharmacology for Natural Products, School of Pharmaceutical Sciences, Kunming Medical University, Kunming, Yunnan, China; 2 Department of Pharmacy, Panzhihua Central Hospital, Panzhihua, Sichuan, China; 3 School of Pharmacy, Dali University, Dali, Yunnan, China; 4 Shandong First Medical University (Shandong Academy of Medical Sciences), Jinan, Shandong, China; 5 The First Hospital of Hebei Medical University, Shijiazhuang, Hebei, China; 6 School of Marine Science and Technology, Harbin Institute of Technology, Weihai, Shandong, China; 7 Department of Pharmaceutics, College of Pharmacy, Jiangxi University of Chinese Medicine, Nanchang, China; 8 Hong Kong Baptist University, Hong Kong, Hong Kong SAR, China

**Keywords:** *Bacillus subtilis* 168, computer simulation, lignosulfonate, MICP, soil erosion in China

## Abstract

**Purpose:**

This study aimed to develop an environmentally friendly microbial-induced calcium carbonate precipitation (MICP) system using *Bacillus subtilis* 168 and calcium lignosulfonate as an alternative carbon and energy source, to reduce ammonia emissions.

**Method:**

We conducted computer simulations and experimental studies, including strain identification and concentration optimization of calcium lignosulfonate and other compounds. Using various detection techniques (SEM, XRD, FTIR, etc.), we evaluated the effectiveness of MICP in concrete and different soil types in China.

**Results:**

The addition of 10 g/L calcium lignosulfonate significantly enhanced calcite formation and improved the cementation ability in Yunnan red soil and Henan yellow soil. The metabolic pathways of *Bacillus subtilis* enzymes (e.g., BsDyP, Ssu) were implicated in lignosulfonate metabolism.

**Conclusion:**

These results demonstrate that calcium lignosulfonate promotes mineralization and can serve as a sustainable carbon and energy source alternative to conventional urea in MICP systems. This approach offers a promising route for concrete reinforcement and soil stabilization by reducing environmental pollution while enhancing calcite formation.

## 1 Introduction

**Figure 1:**
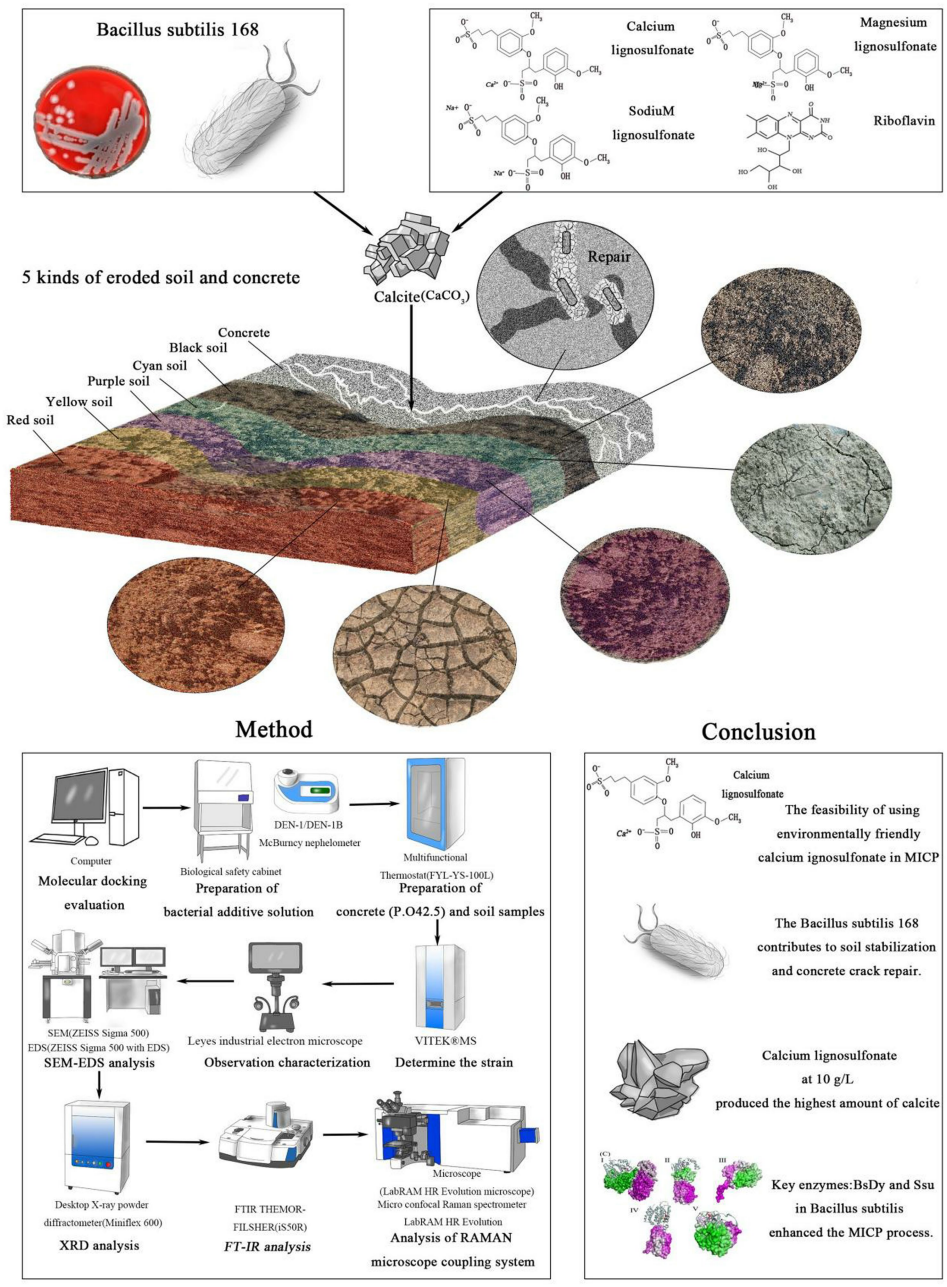
Schematic diagram of this article.

Microbial-induced calcium carbonate precipitation (MICP) has garnered significant attention in recent years due to its potential for ecological and sustainable applications in concrete and soil remediation (Jiang et al., 2023; Zhang et al., 2023; Zhang et al., 2024). This biotechnological process relies on the metabolic activities of microorganisms, particularly *Bacillus subtilis*, to induce the precipitation of calcium carbonate (CaCO_3_), which can solidify soil particles and repair concrete cracks (Cheng et al., 2013; Feng et al., 2021; Jiang et al., 2023). MICP offers an eco-friendly alternative to traditional cementing methods, relying on bacterial metabolism to induce calcium carbonate precipitation (Bhutange and Latkar, 2020; Feng et al., 2021; Raza et al., 2023). Traditionally, MICP systems use urea hydrolysis to facilitate this process, but the resultant ammonia emissions pose environmental risks, including eutrophication and toxicity to aquatic organisms (Bhadiyadra et al., 2024; Chen et al., 2019). In comparison to urea hydrolysis, the use of lignosulfonate significantly minimizes the release of harmful by-products like ammonia (Chen et al., 2019; Su et al., 2022). In response to these challenges, this study proposes an alternative approach, utilizing calcium lignosulfonate as an energy source to drive the MICP process (Grierson et al., 2005; Indraratna et al., 2013). Lignosulfonate water-reducing agents have been used in concrete since the early 1930s and for 40–50 years in China, and when combined with calcium chloride, they form a composite early-strength agent, improving both early and late concrete strength compared to plain concrete. The optimal concentration of calcium lignosulfonate in concrete is approximately 0.3%, with a lignosulfonate concentration range of 8 g/L to 10 g/L, enhancing both the early and late mechanical properties (Jiang et al., 1999; Khan et al., 2022; Singh et al., 2002). Calcium lignosulfonate, a byproduct of the wood pulp industry, serves as a cost-effective and environmentally friendly energy source that minimizes harmful byproducts. The concentration of calcium ions (Ca^2+^) in biocement must remain below 1 mol/L to avoid microbial inhibition, and in this study, the calcium ion concentration used complies with this threshold, ensuring optimal microbial activity. *Bacillus subtilis*, widely recognized as a probiotic for humans and livestock, has been shown to improve buckwheat yield, regulate water quality, and inhibit harmful microorganisms, making it a safe and effective choice for MICP applications in this study. Lignosulfonate’s dual role as an energy source and water reducer further enhances its utility in sustainable construction practices (Witkowicz et al., 2021; Xiang et al., 2022; Yan et al., 2019). Figure 1 shows the MICP mechanism and the role of *Bacillus subtilis* in calcium carbonate precipitation, highlighting the advantages of using calcium lignosulfonate over traditional urea-based systems.

The optimal ratio of calcium lignosulfonate to soil has been found to be around 4%, with a lignosulfonate concentration of 8 g/L to 10 g/L, which enhances soil stabilization and improves mechanical properties (Khan et al., 2022; Lou et al., 2004; Wu et al., 2020). The global soil erosion crisis, exacerbated by unsustainable land use practices and environmental degradation, highlights the need for innovative soil remediation techniques. Studies have demonstrated that lignosulfonates, in low concentrations, can act as environmentally friendly soil additives, improving soil properties without harming animal husbandry, with evidence supporting their safe use as feed additives for poultry and lambs (Cirne et al., 2020; Corey et al., 2014; Kang et al., 2022; Xu, 2018). In regions like China, where extensive soil erosion and degradation occur, MICP offers a promising solution. Various soil types, such as Yunnan red soil, Henan yellow soil, and Sichuan purple soil, are highly susceptible to erosion, threatening agricultural productivity and environmental stability. At the same time, concrete infrastructure worldwide faces durability challenges due to cracking and degradation, which reduces its lifespan and increases maintenance costs (Charalambidi et al., 2021; Tang et al., 2016; Zhou et al., 2021). As urbanization accelerates, the demand for effective, sustainable solutions to improve soil and concrete stability grows. Implementing MICP with environmentally friendly calcium lignosulfonate could provide a viable, eco-conscious alternative to conventional methods, offering enhanced mechanical properties for soil stabilization and concrete repair without the environmental burdens associated with urea hydrolysis-based systems. Moreover, the reduced ammonia emissions align with global sustainability goals, mitigating environmental risks (Bhadiyadra et al., 2024; Fahimizadeh et al., 2022; Tao et al., 2025).

In this study, we employed a range of analytical techniques to evaluate the effectiveness of MICP in concrete and soil stabilization. A comprehensive methodology was implemented, including microbial strain identification, calcium lignosulfonate concentration optimization, and characterizations using scanning electron microscopy (SEM), X-ray diffraction (XRD), Fourier-transform infrared spectroscopy (FTIR), and Raman microscopy. These techniques enabled us to observe the crystallization of calcium carbonate within the treated samples and assess the mechanical improvements imparted by the MICP process. Additionally, molecular docking simulations provided insights into the metabolic pathways of *Bacillus subtilis*, particularly focusing on the role of enzymes such as BsDyP and Ssu in lignosulfonate metabolism (Kim et al., 2015; Rana et al., 2023; Xu et al., 2006). The results from both laboratory experiments and computational models offered a thorough understanding of the interactions between the microorganisms and the calcium lignosulfonate, leading to optimized conditions for calcium carbonate precipitation in various soil types and concrete matrices. The interaction between *Bacillus subtilis* and calcium lignosulfonate also enhances the mechanical strength of treated materials, particularly in soil stabilization (Metuge and Senwo, 2024; Prasetyo et al., 2010; Wu et al., 2020).

The purpose of this article is to explore the potential of calcium lignosulfonate as an alternative energy source for MICP, and creatively apply calcium lignosulfonate to MICP process, specifically its applications in concrete crack repair and soil stabilization, breaking through the limitations of traditional urea system. By demonstrating the effectiveness of calcium lignosulfonate in enhancing calcite production while minimizing environmental impact, this study aims to present a sustainable and scalable solution for addressing challenges in soil erosion and concrete degradation. Using calcium lignosulfonate as MIPC alternative energy technology proposed in this paper provides a solution to solve the contradiction between construction engineering development and environmental sustainable development, which not only solves the problem of ammonia pollution in the process of applying MICP technology, but also creates a new value chain, providing a demonstration case for realizing the industrialization of bioremediation technology. The inclusion of *Bacillus subtilis* as a non-pathogenic strain further supports its safe application in environmental biotechnology.

## 2 Materials and methods

**Figure 2:**
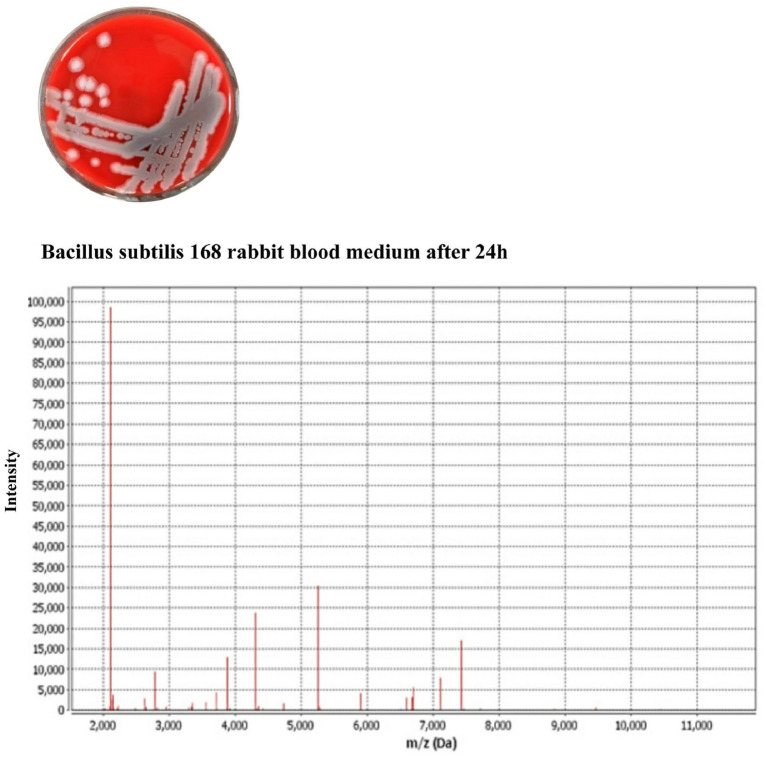
Identification of *Bacillus subtilis* 168.

In this study, freeze-dried *Bacillus subtilis* 168 strains were used to induce microbial-induced calcium carbonate precipitation (MICP) in soil and concrete samples. Soil samples from Yunnan (red soil), Henan (yellow soil), Sichuan (purple soil), Jiangsu (cyan soil), and Heilongjiang (black soil) were prepared, alongside Portland cement P.O 42.5 for concrete experiments. Calcium lignosulfonate, magnesium lignosulfonate, sodium lignosulfonate, and riboflavin were tested as energy sources for bacterial metabolism. After preparing bacterial additive solutions containing *Bacillus subtilis* 168, calcium chloride (CaCl₂) was added to serve as a calcium ion source. The bacterial additive solutions were inoculated into both soil and concrete samples, and the experiments were conducted in a controlled incubator for 28 days. Various concentrations of calcium lignosulfonate (2, 4, 6, 8, and 10 g/L) were tested to optimize MICP activity. The experimental apparatus included SEM, EDS, XRD, FTIR, and Raman microscopy to evaluate the microstructural changes, calcium carbonate precipitation, and elemental compositions of the samples. To further investigate the interaction between calcium lignosulfonate and *Bacillus subtilis*, molecular docking analysis was performed to explore the metabolic pathways involved. The results were analyzed through microscopic and spectroscopic methods to determine the extent of MICP, with a focus on calcium carbonate crystallization and mechanical improvements in both soil and concrete samples. Detailed methods are shown in Figure 2.

### 2.1 Materials

Freeze-dried powder of *Bacillus subtilis* 168 strains (American strain source, 0-generation bacteria in China) were obtained from Shanghai Preservation Biotechnology Center (China). We purchased experimental specimen soil (red soil from Yunnan Province, yellow soil from Henan Province, purple soil from Sichuan Province, cyan soil from Jiangsu Province, and black mud from Heilongjiang Province, China) from Suzhou Didon Science and Education Equipment Co., Ltd. (China). Experimental cement (belonging to the grade of experimental equipment cement) is a Chinese standard P.O42.5 Portland cement purchased from Zhu cheng Yang chun Cement Co., Ltd. (China). We bought a 48-well culture plate (with a diameter of 17 mm and a depth of 7 mm) from Haiying Experimental Equipment Co., Ltd. in Haimen City (China) and curved glass dishes (diameter 200 mm, height 20 mm) purchased from Yan cheng Zhi xue Experimental Equipment Co., Ltd. (China).

### 2.2 Experiment reagent

Calcium lignosulfonate [≥98.0%; Hefei BASF Biotechnology Co., Ltd. (China)]; Sodium lignosulfonate [≥98.0%; Hefei BASF Biotechnology Co., Ltd. (China)]; Magnesium lignosulfonate [≥99.9%; Wu han Feng zhu Chemical Technology Co., Ltd. (China)]; Vitamin B2 (riboflavin) [≥98%; National Pharmaceutical Group Chemical Reagents Co., Ltd. (China)]; anhydrous calcium chloride [≥96%; McLin Reagents Co., Ltd. (China Division)]. nutrient broth medium (composition (g/L): peptone 10.0, sodium chloride 5.0, beef extract 3.0, glucose 4.0, pH 7.2 ± 0.4) was purchased from Hangzhou Binhe Microbial Reagents Co. All chemicals and biological reagents are reagent grade purity in line with experimental standards.

### 2.3 Experimental apparatus

DEN-1/DEN-1B Maxwell Turbidimeter (China), Multifunctional Thermostat (FYL-YS-100L) (China), VITEK^®^MS (France), FTIR THEMOR-FILSHER (iS50R) (USA), Desktop X-ray Powder Diffractometer (MiniFlex600) (Japan), Microscopic Confocal Raman Spectrometer LabRAM HR Evolution (France), Microscope (LabRAM HR Evolution comes with a microscope) (France), SEM (ZEISS Sigma500) (Germany), Eyes Industrial Electron Microscope (1000×) (China), EDS (ZEISS Sigma 500 with energy spectrometer EDS) (Germany), Biological Safety Cabinet (China).

### 2.4 Molecular docking evaluation

#### 2.4.1 Ligand preparation

Select substances that can provide energy for bacteria as the compounds in this experiment among soil and concrete additives. These compounds can affect bacterial MICP, and we use these compounds as docking ligands. From the PubChem database^1^ Retrieve and download ligand structures from the ZINC database,^2^ ChemDraw20.0 software was used to minimize the energy with MMFF94. The ligand was retained for molecular docking.

#### 2.4.2 Protein preparation

In this study, MICP used *Bacillus subtilis* 168 (probiotic of animals and plants). From the literature, we searched the metabolic processes and enzymes involved in *Bacillus subtilis* 168. Then we obtained the corresponding protein crystal structure from the PDB database.^3^ Reduce and simplify protein energy with Discovery Studio 2019 software. We uploaded the ligands and targets to the CB-Dock website (CB-Dock: An accurate protein-ligand blind docking tool^4^) for molecular docking. The molecular docking binding energy was visualized by Origin 2022 software.

#### 2.4.3 Analysis of lignosulfonate metabolism

In this experiment, lignosulfonate was selected due to its compliance with the standards for concrete additives in China. The primary focus of this study was to investigate the metabolic pathways involved in the degradation of lignin and sulfonates by *Bacillus subtilis*. Using the KEGG database, the target proteins associated with this metabolism were identified and analyzed. The SsuACBD gene cluster was employed as the enrichment background to further investigate the sulfonic acid sulfur metabolic pathway and the relevant enzymes involved.

### 2.5 Experimental material pretreatment

#### 2.5.1 Preparation of bacterial additive solution

After activating the freeze-dried *Bacillus subtilis* 168, it was inoculated on the Blood Agar Plate (beef extract powder 10 g/L, sodium chloride 5 g/L, agar 15 g/L, defibrated sheep or rabbit blood 80 mL/L) in a biosafety cabinet, with a final pH of 7.4 ± 0.2. Bacillus subtilis 168 was nutritional after growing at 31 °C for 24 h. *Bacillus subtilis* 168 was prepared into 4.0 OD (12.0 × 10^8^ CFU/mL) bacterial solution, and 2.2 g nutrient broth and 0.095 mol/L calcium source were added (Figure 4C) (Park et al., 2012). In this experiment, CaCl_2_ (110.984) 1.543 g was added to configure the solution (CaCl_2_:10.543 g/L). After the oscillation was uniform, we configured it into 100 mL of biological additives with pH 8.0. According to the demand, more additives of the same proportion can be prepared. The concrete ratio: 25 g portland cement added 8 mL distilled water, in the concrete is just solidified state add bacterial additives (Park et al., 2012).

#### 2.5.2 Preparation of concrete (P.O42.5) and soil samples

This experiment configured concrete and soil in 48-well plates for easy observation. The concrete experiment was seven groups. Group A was the blank group, Group B was the bacterial control group, and C-F added different concentrations of calcium lignosulfonate, magnesium lignosulfonate, sodium lignosulfonate, and riboflavin based on the bacterial group. Group G was CaCl_2_ (This study converted the calcium source control group into 0.42 g/L, 0.84 g/L, 1.26 g/L, 1.68 g/L, 2.10 g/L according to the calcium ion concentration of calcium lignosulfonate. Since calcium ion may affect the crystallization result of calcium carbonate, we add CaCl_2_ with the same Molar mass calcium ion concentration as the calcium ion control group.) added bacterial group. The soil experiment consists of six groups. Group A is a blank group, Group B is a bacterial control group, and Group C–F adds different concentrations of calcium lignosulfonate, magnesium lignosulfonate, sodium lignosulfonate, and riboflavin to the bacterial group. Please refer to Supplementary Table S1 for detailed information. Due to the excellent demand for Raman spectroscopy samples, this experiment repeated Group C experiments with curved glass dishes. The concrete and soil in this experiment were simulated natural environment, and there was no ultraviolet pretreatment. We inoculated the concrete and soil with *Bacillus subtilis* 168 samples in a multifunctional incubator for 28 days (Culture temperature of 31 °C, humidity of 40% RH). Since the Raman study requires a larger amount of material, a larger size Petri dish was selected for this experiment to repeat the above Group C experiment for the Raman experimental group, as shown in Supplementary Table S2.

The five typical Chinese soil types used in this study present distinct differences in texture, mineral composition, organic matter content and basic physicochemical properties, all of which can influence bacterial colonization, Ca^2+^ availability and CaCO₃ crystallization during MICP.

Yunnan red soil is highly weathered, rich in iron and aluminum oxides, acidic, and characterized by low cation exchange capacity, rendering it highly vulnerable to water erosion.

Henan yellow soil is loess-derived, dominated by silt with low organic carbon and a loose structure, making it prone to erosion and structural instability.

Sichuan purple soil forms from purple sandstone and shale, is mineral-abundant and near-neutral to weakly acidic; although relatively fertile, it exhibits weak aggregate stability and is easily degraded.

Jiangsu cyan soil is a typical paddy soil with high clay content and low permeability, often containing reduced iron and manganese compounds.

Heilongjiang black soil suffers severe erosion, exhibits compromised structural integrity and reduced resistance to disturbance, rendering it highly vulnerable to external attack.

These inherent soil properties help explain the varied performance of MICP remediation observed among different soil samples.

### 2.6 Through VITEK^®^MS determines the strain

*Bacillus subtilis* 168 bacteria (USA) were activated and placed on the slide. After the colony was formed, we added the matrix solution (VITEK MS-CHCA). After air-drying, the slide was inserted into the VITEK MS system for detection. The laser frequency is 200 Hz. The sample was analyzed in linear positive ion mode with a mass-to-charge ratio of 1,500 to 12,000 Da. External quality calibration was performed using TOF (TOF-MS) (BioMerieux). After the spectral acquisition, microbial identification was achieved by comparing the Vitek^®^MS spectral database.

### 2.7 Industrial electron microscope observation characterization

The MICP biological precipitation of concrete and soil and the condensation of soil glue were observed under the Leyes industrial stereoscopic electron microscope (China).

### 2.8 SEM-EDS analysis

We used scanning electron microscopy (ZEISS Sigma 500) (Germany) to observe the surface microstructure of groups A, B, and C of concrete. The concrete surface is coated with gold mist and scanned at magnifications of 5,000×, 10,000×, 20,000×, and 50,000×. Energy measurement was performed using an X-ray energy chromatograph (EDS). The spectrometer detector: (SDD) electric refrigeration detector, ultra-thin window design, 30 mm2 effective area; the energy resolution (100 kcps) does not exceed 129 eV (Mn-Ka), and the elemental composition (%) was obtained.

### 2.9 XRD analysis

The crystal form of the deposited calcium carbonate was identified by X-ray diffractometer XRD (MiniFlex600) (Japan). Remove the residue from the graphite rod and manually grind the material to prepare the sample. The measurements were carried out at room temperature using CuK^α^ radiation on a diffractometer with a Bragg–Brentano geometry. The test conditions were Cu target radiation (*λ* = 0.15406 nm), a working current of 40 mA, a working voltage of 40 kV, a scanning range of 10–90, a scanning rate of 5 min, and a step size of 0.02. Fine structure detection slow scan (Bhadiyadra et al., 2024). X-ray powder diffraction patterns were collected over an hour.

### 2.10 FT-IR analysis

The FT-IR spectra were recorded using FTIR THEMOR-FILSHER (iS50R, United States). The resolution in the transmission mode was ± 4 cm^−1^, and all spectra were recorded in the 4,000 to 400 cm^−1^. The concrete fine powder sample was mixed with pure KBr at a ratio of 1: 50 and pressed into a disc of about 1 mm thickness. Due to the complexity and particularity of the composition of the soil itself, to avoid the water absorption peak generated by KBr water absorption interfering with the soil test results, the soil powder was detected by ATR-FTIR.

### 2.11 Analysis of Raman microscope coupling system

To confirm the detection results of XRD and judge the difference of crystal seeds, the experiment was carried out by coupling the LabRAM HR Evolution (France) instrument produced by HORIBA Jobin Yvon with the BX41 microscope (Olympus, Hamburg, Germany) and the excitation and collection were carried out under the 10×/N.A. = 0.25 objective. The system has 300 I/mm and 1800 I/mm gratings, providing liquid N to collect spectra with different ranges and resolutions. The excitation wavelength is 532 mm laser.

## 3 Result

### 3.1 Additive compounds beneficial for MICP bacterial metabolism in literature retrieval

**Figure 3:**
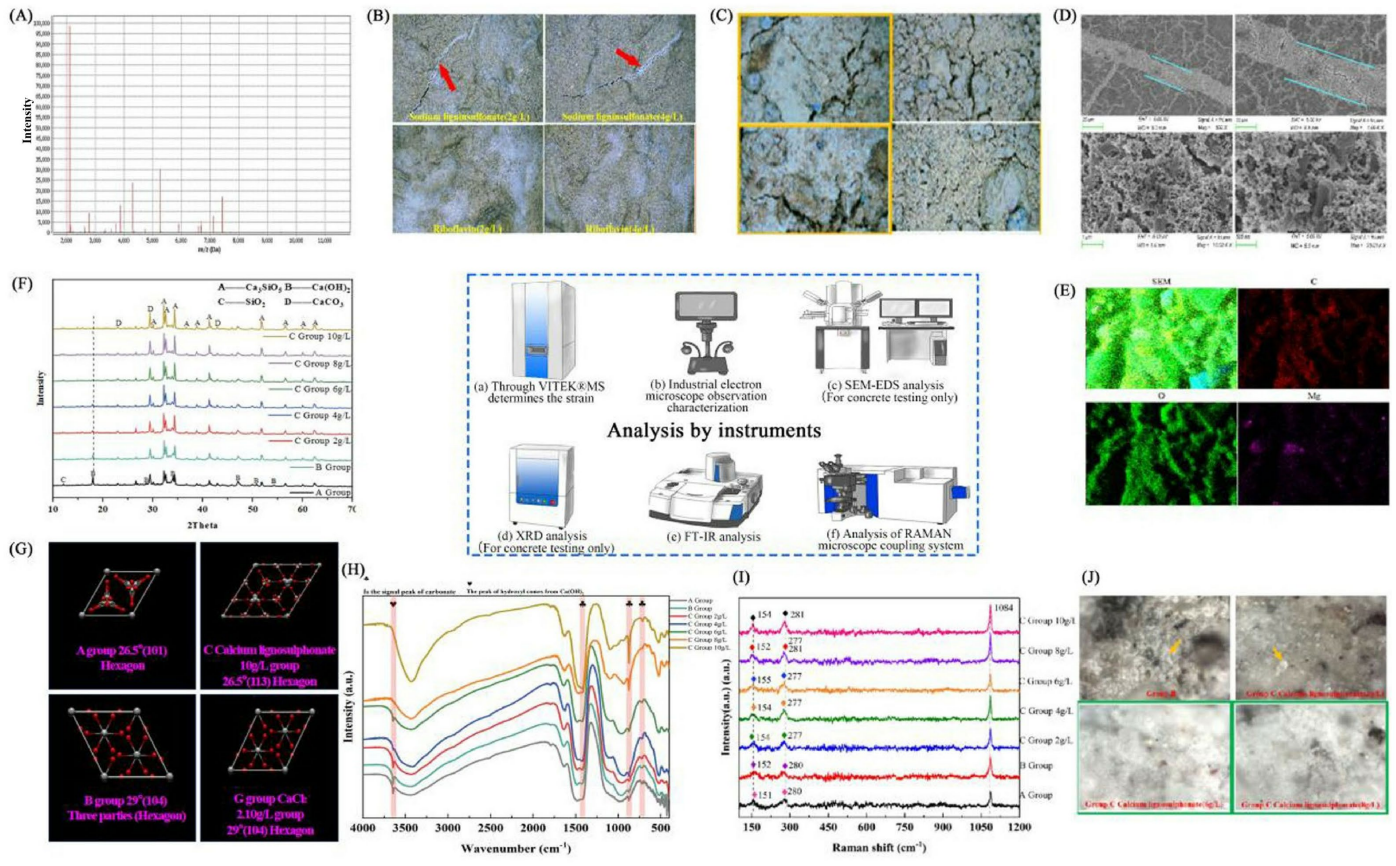
Analysis by instruments. **(A)** Identification of *Bacillus subtilis* 168. (By using VITEK^®^MS). **(B)** *Bacillus subtilis* 168 concrete sample. (By using Eyes Industrial Electron Microscope). **(C)** Picture of *Bacillus subtilis* 168 sample. (By using Eyes Industrial Electron Microscope). **(D)** Sample diagram of *Bacillus subtilis* 168 detected by SEM. **(E)** Sample diagram of *Bacillus subtilis* 168 detected by SEM and EDS. **(F)** Sample diagram of *Bacillus subtilis* 168 detected by XRD. **(G)** Sample diagram of *Bacillus subtilis* 168 detected by XRD. **(H)** Sample diagram of *Bacillus subtilis* 168 detected by FT-IR. **(I)** Sample diagram of *Bacillus subtilis* 168 detected by Raman. **(J)** Sample diagram of *Bacillus subtilis* 168 detected by Raman.

After a comprehensive review of the literature, China’s soil management policy, and China’s concrete additive policy, Lou’s et al. (2004) study have shown that calcium lignosulfonate positively promotes bacterial growth and reproduction at 8 g/L. It is greater than 8 g/L. Lignosulfonate also improves microbial adhesion to soil particles, enhancing the overall bio-cementing effect in MICP applications. The promotion effect of 10 g/L is second only to 8 g/L. Magnesium lignosulfonate positively promoted the growth and reproduction of bacteria at 6 g/L (Lou et al., 2004). Sodium lignosulfonate promoted the hydrolysis and fermentation of *Bacillus subtilis*. When the addition amount was 1 g/L, viable bacteria increased to 2.10 × 10^9^ CFU/ML. Studies have shown that the presence of riboflavin can stimulate the metabolism of *Bacillus subtilis* (Chu et al., 2022; Wei et al., 2022). The compound is shown in Figure 3A, Related protein analysis is shown in Table 1.

**Figure 4:**
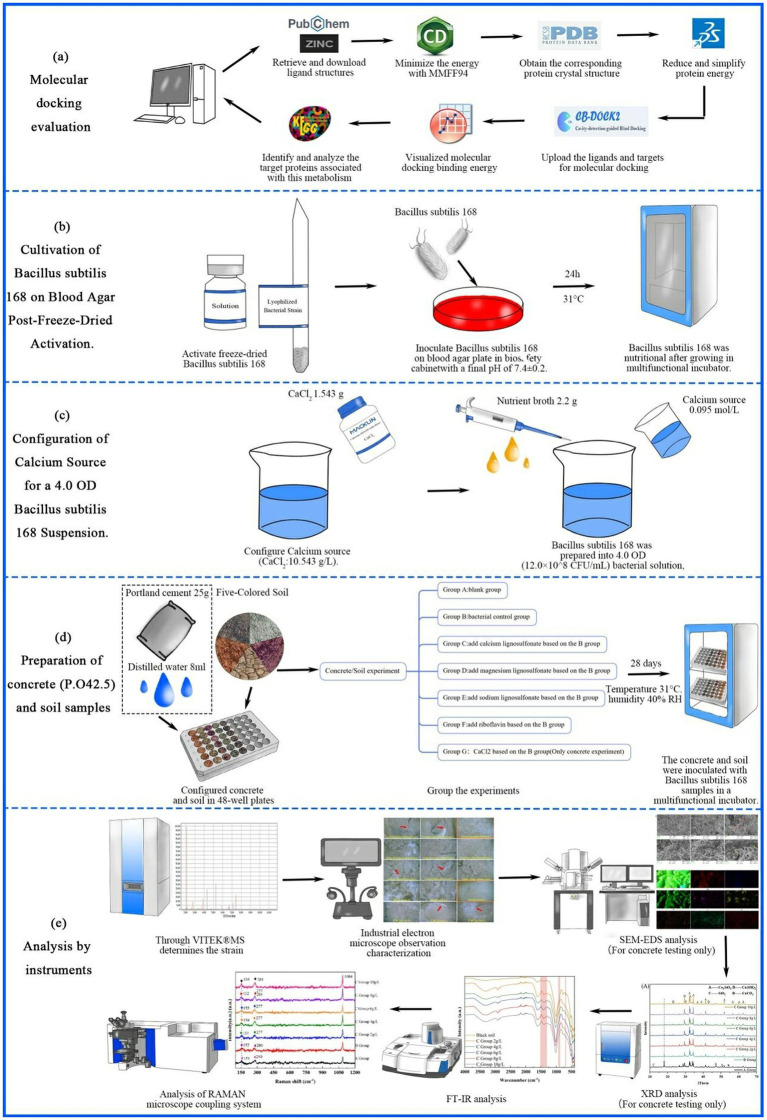
Schematic overview of the experimental workflow. **(a)** Molecular docking evaluation of *Bacillus subtilis* 168 enzymes with calcium lignosulfonate. **(b)** Activation and cultivation of freeze-dried *Bacillus subtilis* 168 on blood agar plates. **(c)** Preparation of 4.0 OD bacterial suspension with calcium source. **(d)** Preparation of concrete and soil samples and grouping of experiments. **(e)** Multi-technique characterization workflow (VITEK®MS, industrial electron microscopy, SEM-EDS, XRD, FT-IR, and Raman analysis).

**Table 1:** Protein name and gene name.

Protein name	Gene name	Enzyme name
7pl0	B4417_2151, CFD21_01545	Deferrochelatase/peroxidase (Bs DyP)
7dlk	efeB, gS595_16230	Deferrochelatase/peroxidase (DyPs)
5y0Q	B4417_3650	LI gASE
A0A6M4JgP3	ssuD	ssuD
4kjr	yfkE, BSU07920, chaA	cation exchan ger YfkE
7pkx	B4417_2151, CFD21_01545	Deferrochelatase/peroxidase (Bs DyP)
1gSK	CotA	SPORE COAT PROTEIN A
P40402	ssuD	ssuD
A0A6M3Z7U8	HIR78_03255, ydhB	Probable membrane transporter protein
A0A6M3Z8S0	ssuA	ssuA
4kjs	CotA	CotA
O05493	ydhB	Probable membrane transporter protein
4YVU	cotA, pig, BSU06300	CotA
4Q89	cotA, pig, BSU06300	CotA
1ih8	outB	NH (3)-DEPENDENT NAD (+) synthetase
A0A6M4JM68	HIR78_06840	Probable membrane transporter protein
A0A6M3ZgB5	HIR78_18780	Probable membrane transporter protein
P18318	ureF	Urease accessory protein UreF
O34578	yjnA	Probable membrane transporter protein YjnA
P07788	CotA	Laccase
A0A6M4JNS2	HIR78_15590	Probable membrane transporter protein
O32134	yunE	Probable membrane transporter protein YunE
P77837	ureC	Urease subunit alpha
P97027	ssuB	Aliphatic sulfonates import ATP-binding protein SsuB
P71035	ureB	Urease subunit beta
P75030	ureA	Urease subunit gamma
P54437	yrkJ	Probable membrane transporter protein YrkJ
O34430	ytnM	Probable membrane transporter protein YtnM
A0A6M4JL10	HIR78_17080	Probable membrane transporter protein

### 3.2 Analysis of molecular docking results

Lower binding energy between a ligand and receptor increases the likelihood of interaction and enhances binding efficiency. The sulfonates examined in this study contain various cations, but the parent sulfonate structure is common across the different compounds. For the molecular docking analysis, we selected the same anionic sulfonate parent (without cations) as depicted in Figure 3A, and docked it against the target proteins listed in Table 1.

The binding energies for the target macromolecular proteins were all below −5.0 kJ/mol, suggesting potential metabolic activity related to *Bacillus subtilis* 168. A heatmap illustrating the molecular docking binding energies is presented in Figure 3B, with proteins involved in similar metabolic processes sharing color and shape codes. Figure 3C depicts the binding models between the sulfonate matrix and various protein types, while the specific protein types are detailed in Table 2.

**Table 2:** Key protein names and key gene names.

Protein name	Gene name	Enzyme name
7pl0	B4417_2151, CFD21_01545	Deferrochelatase/peroxidase (Bs DyP)
5y0Q	B4417_3650	LIgASE
A0A6M4JgP3	ssuD	ssuD
4kjr	yfkE, BSU07920, chaA	cation exchanger YfkE
1gSK	CotA	SPORE COAT PROTEIN A

Sulfonate parent (without cations) as depicted in Figure 3A, and docked it against the target proteins listed in Table 1. The binding energies for the target macromolecular proteins were all below −5.0 kJ/mol, suggesting potential metabolic activity related to *Bacillus subtilis* 168.

A heatmap illustrating the molecular docking binding energies is presented in Figure 3B, with proteins involved in similar metabolic processes sharing color and shape codes. Figure 3C depicts the binding models between the sulfonate matrix and various protein types, while the specific protein types are detailed in Table 2. Complementary characterization of the bacterial strain and experimental samples, including stereomicroscopic images (Figure 3D), SEM-EDS elemental mapping (Figure 3E), XRD patterns (Figures 3F,G), and FTIR/Raman spectra (Figures 3H–J), further supports these findings by validating the presence of CaCO_3_ precipitates and mineral structural changes induced by the MICP process.

These strong binding interactions help explain the improved bacterial growth, increased CaCO₃ precipitation, and better soil cementation observed in the calcium lignosulfonate groups.

### 3.3 Lignosulfonate metabolic pathway of *Bacillus subtilis* 168

*Bacillus subtilis* 168 metabolizes lignin mainly through five types of enzyme catalytic pathways: Deferrochelatase/peroxidase BsDyP; ssu enzyme pathway; Ca^2+^ channel enzyme; Laccase (*Bacillus subtilis* COTA); Lip (lipase). The bacterial micro-metabolism of “*Bacillus subtilis* 168-10 g/L calcium lignosulfonate” was shown in Figure 3D.

#### 3.3.1 Deferrochelatase/peroxidase *Bacillus subtilis* BsDyP

It has been reported that the genome analysis of *Bacillus subtilis* SCK6 showed that the ligninolytic enzyme was composed of DyP and laccase, which was annotated based on the peroxide alkali and CAZy database (Qin et al., 2021). It has been reported that DyP can act on various substrates, including lignin-derived phenols and non-phenolic compounds, synthetic high-redox potential anthraquinones, and azo dyes (de Eugenio et al., 2021; Qin et al., 2021).

This catalytic capability supports the efficient utilization of calcium lignosulfonate as a carbon and energy source in our MICP system, consistent with the increased calcite formation seen in SEM and XRD.

#### 3.3.2 Ssu enzyme

The genetic background for using sulfonates has been identified in the past few years. A group of genes involved in using sulfonates, called ssu genes (sulfate sulfur utilization), were described for *Bacillus subtilis*. In all species, the ssu gene is organized as an operon responsible for the specific uptake and utilization of aliphatic sulfonates (van der Ploeg et al., 1998). The ssu operon consists of three genes encoding ABC-type transporters ssuA, ssuB, and ssuC, and a ssuD gene that directs the synthesis of reduced flavin mononucleotide (FMNH). The lignosulfonate precursor is converted to sulfite by two alkane sulfonate monooxygenases, ssuD, and ssuE, and then converted to volatile compound hydrogen sulfide (H_2_S) (Eida et al., 2020). In this paper, KEGG searches metabolic pathways (Figure 4A).

This sulfonate-specific metabolic pathway accounts for the near absence of ammonia release in our system, distinguishing it from urea-based MICP and supporting our environmentally friendly design.

#### 3.3.3 Ca^2+^ channel

In 1997, the whole genome sequence of *Bacillus subtilis* 168 was determined (Cheng et al., 1996). Only one Ca^2+^ member: cation antiporter (CaCA) family was found in this genome: yfkE (Chung et al., 2001). *Bacillus subtilis* 168 and yfkE. Studies have shown that it is involved in the production of lignocellulolytic enzymes by regulating the changes of Ca^2+^ in *Bacillus subtilis* cells (Liu et al., 2022). The precipitation of CaCO_3_ in MICP is closely related to this channel.

Modulation of intracellular Ca^2+^ homeostasis via this channel promotes CaCO₃ nucleation and crystal growth, consistent with the denser, more stable calcite observed in the 10 g/L calcium lignosulfonate groups.

#### 3.3.4 Laccase (*Bacillus subtilis* COTA OCueO) laccase

Through the analysis of the literature related to HPLC, the possible metabolites of lignin structure in laccase metabolism, there is a team analysis of OcCueO incubated with eight different lignin preparations (Including organic solvent lignin, alkali lignin, lignosulfonate and industrial kraft paper lignin) Only in the case of lignosulfonate, low molecular weight products were detected by HPLC analysis. In the case of Ca-lignosulfonate, a new peak was formed at 27 min retention time, and its retention time and mass spectrum were matched with vanillic acid (m/z 169 MH, 191 MNa) (Granja-Travez et al., 2018). The phthalic acid ester derivatives were detected in the HPLC re-detection of lignin decomposition by *Bacillus subtilis* and in the fungal peroxidase degradation of lignosulfonate (Yadav and Chandra, 2015).

The generation of lignin-derived small-molecule metabolites such as vanillic acid stimulates bacterial metabolism and further promotes CaCO₃ precipitation, consistent with our macroscopic and microscopic observations.

#### 3.3.5 Lip (lipase)

Ligninase (LiP) seems to be a catalyst for cutting non-phenolic compounds with high redox potential with carbon–carbon bonds, carbon–oxygen bonds, and oxidative bond cleavage. The multiple abilities of LiP are triggered by its stability in the degradation of xenobiotics and non-phenolic compounds (Shin and Lee, 1999; Singh et al., 2021).

### 3.4 Analysis of sulfonate pathway in KEGG

As shown in Figures 3D, 4A, the analysis of the KEGG pathway reveals that lignin sulfonates, specifically alkane sulfonates, undergo a stepwise metabolic process. Initially, these compounds are oxidized and reduced to alkane sulfonates through the ssuACB system. Following this, they are further oxidized and reduced to sulfite via the activity of ssuD (1.14.14.5), a key enzyme in the pathway. The final step involves the oxidation and reduction of sulfite to sulfate, facilitated by the 1.8.3.1 enzyme system. These metabolic steps highlighting the role of key enzymes in the transformation of sulfonates into sulfate.

This sequential metabolism of sulfonates maintains a steady, non-toxic nutrient supply that supports consistent CaCO₃ deposition in concrete and various soil types.

### 3.5 Determine whether MICP can be used under natural conditions and the importance of various conditions

**Figure 5:**
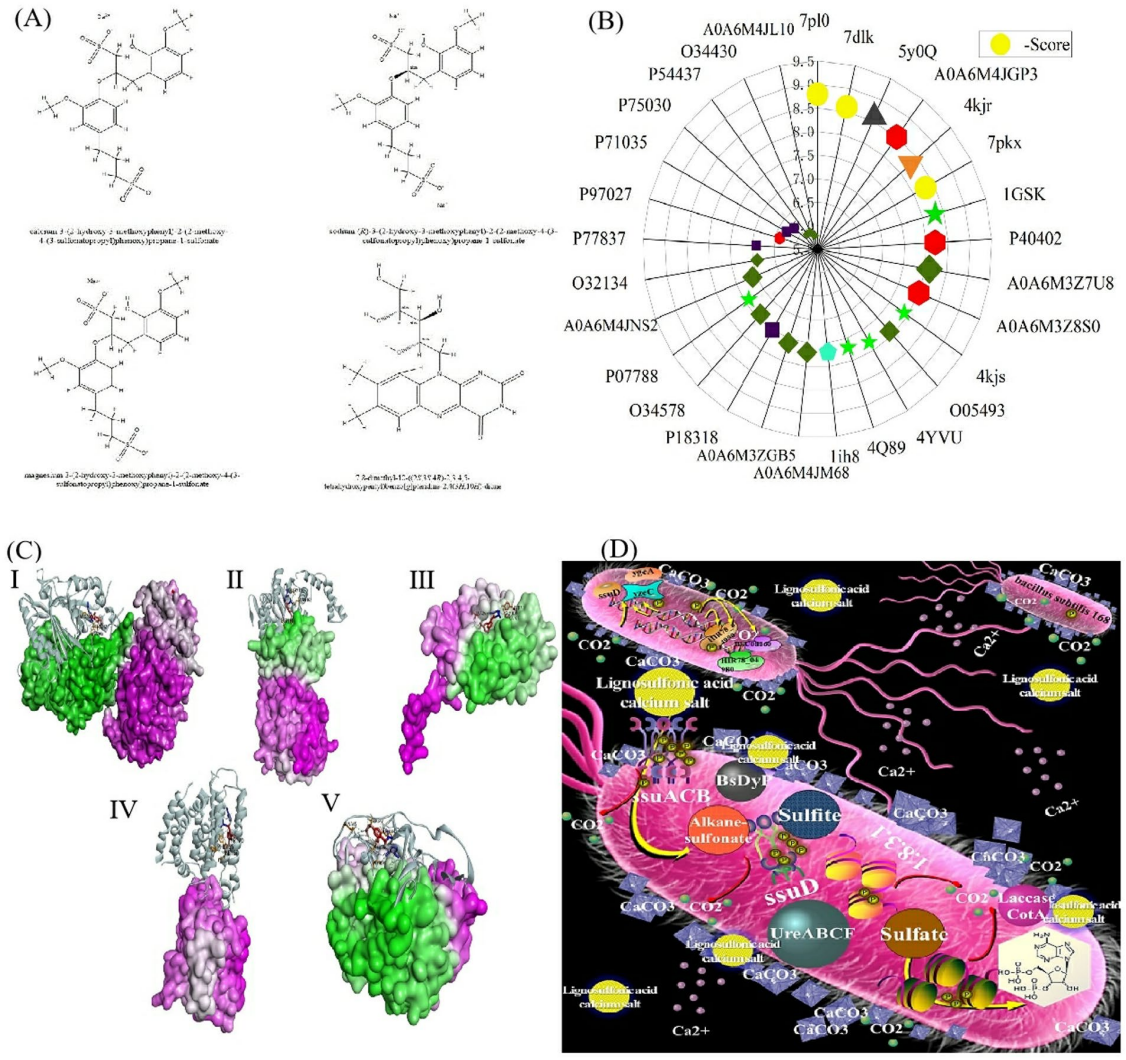
Metabolic process analysis of *Bacillus subtilis* 168. **(A)** The chemical structure of four nutrients. **(B)** Affinity score chart. **(C)** Chimeric model of maternal molecules and related metabolic enzyme proteins. **(D)** Schematic diagram of lignin sulfonate metabolism in *Bacillus subtilis* 168. I: Protein 7pl0, II: Protein 5y0q, III: Protein A0A6M4JGP3, IV: Protein 4kgr, V: Protein 1gsk.

In order to assess whether microbial-induced calcium carbonate precipitation (MICP) can be effectively applied under natural conditions, a multi-layer perceptron (MLP) network was developed. This model helps to visualize and evaluate the importance of several conditions necessary for MICP to function effectively. The network consists of two hidden layers, each with five neurons. Using the mlpcla model, the plotnet function was employed to visualize the network structure and to observe the connections between the neurons. In the network, red lines indicate positive effects on MICP suitability, while gray lines represent negative effects. The thickness of these lines corresponds to the magnitude of the weight or importance assigned to each variable. This MLP network enables the calculation and visualization of the relative importance of each independent variable in predicting the success of MICP under natural conditions. According to our findings, the three most significant factors influencing the suitability of MICP for natural crack repair are (1) whether the nutrients used contribute to environmental pollution, (2) the amount of calcium carbonate (CaCO_3_) crystals produced, and (3) whether the bacterial strain employed is pathogenic (Figure 5B). Additionally, the prediction accuracy of the MLP classifier was assessed using a confusion matrix. This matrix provides a visual representation of the model’s ability to correctly classify samples. As shown in Figure 5C,II, the classifier effectively predicted the MICP outcome for crack repairs. As shown in Figure 5C,III, achieving 100% accuracy for certain conditions.

### 3.6 Confirmation of *Bacillus subtilis* 168 types

To confirm the correct strain, the surface morphology of the strain was observed. Usually when growing in liquid media, the surface of the colony is rough, opaque, gray, and wrinkled, which is consistent with *Bacillus subtilis* 168 (Zeigler et al., 2008). Instrument detection was used to confirm the type of bacteria. VITEK^®^MS and Vitek MS showed *Bacillus subtilis* 168 with 99% confidence. The result is shown in Figure 2.

### 3.7 Characterization of concrete

The surface morphology of both concrete and soil samples was examined using an industrial electron microscope (China) at a magnification of 1,000×. As riboflavin, known for its yellow-green fluorescence, was used in the experiments, the samples’ color intensified as the concentration increased, turning a darker yellow in Group F. In this study, only Group G was added to the concrete due to the complexity of soil compositions and the limited number of characterization tests performed. The comparison between Group C and Group G was more clearly observed through the concrete characterization, as concrete typically contains fewer elements and has a smoother surface. Additionally, CaCl_2_, while effective, is costly and extensively used in environmental applications, contributing to a significant economic burden for government projects.

### 3.8 Representation appears

Laboratory P.O42.5 concrete (Figure 5): A fascinating phenomenon is that cracks accidentally generated by calcium lignosulphonate (2 g/L, 4 g/L) in Group C, sodium lignosulfonate (2 g/L, 4 g/L, 8 g/L) in Group E and CaCl_2_ (0.84 g/L, 1.26 g/L, 1.68 g/L) in Group G are filled with biologically precipitated CaCO_3_ (marked with a red arrow). Group C calcium lignosulphonate (6 g/L, 8 g/L, 10 g/L), Group F riboflavin (6 g/L), Group G CaCl_2_ (2.10 g/L) concrete surface covered with biological precipitation CaCO_3_, in which the precipitated CaCO_3_ covered with calcium lignosulfonate in Group C is the thickest, white CaCO_3_ covered the surface of the whole concrete sample (marked with orange box). The above results were verified in the following SEM and XRD characterization.

**Figure 6:**
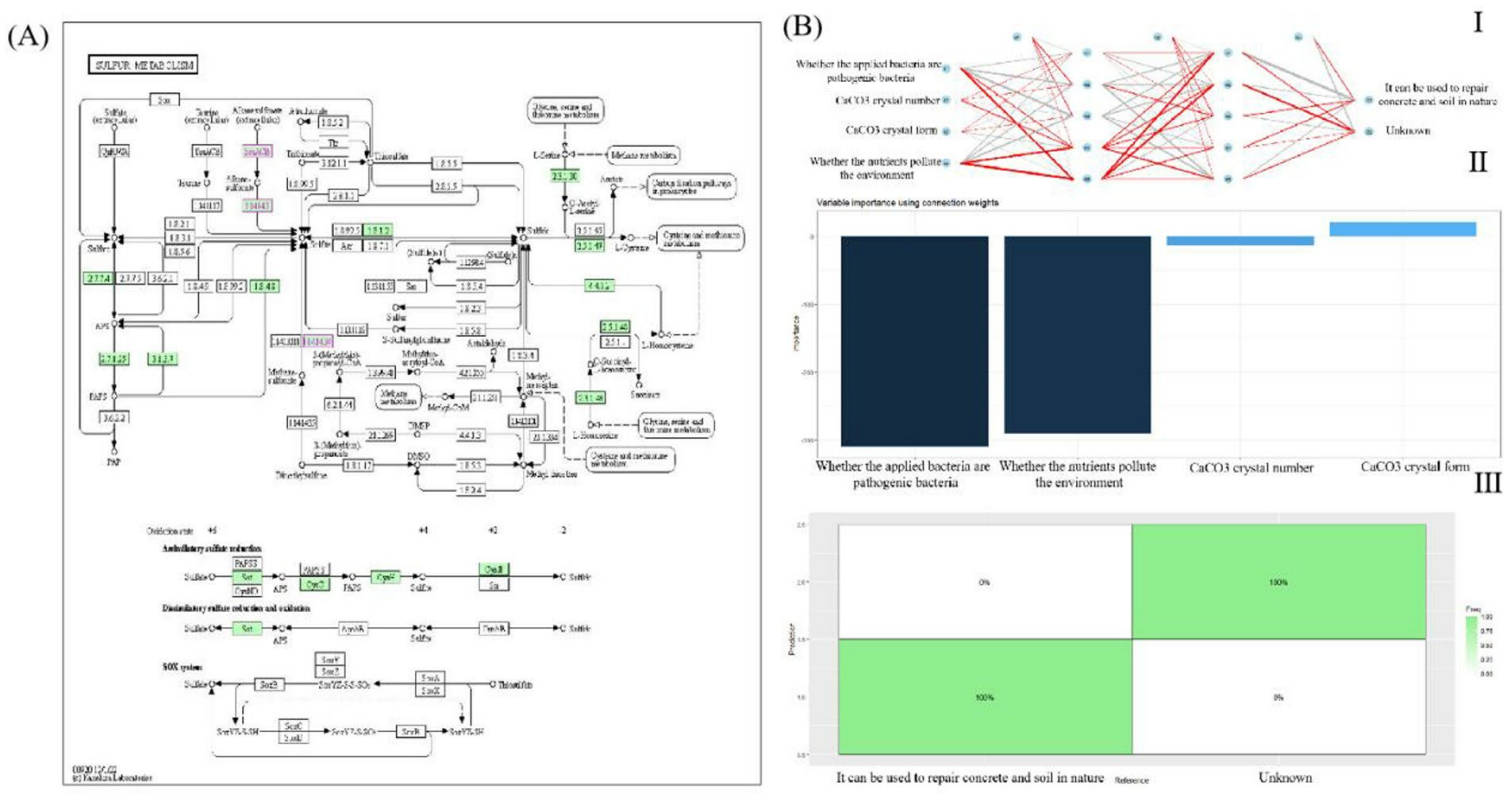
*Bacillus subtilis* 168 ssu gene metabolism and possibilities for MICP technology. **(A)** Sulfonic acid metabolism in the ssu system of *Bacillus subtilis* 168. **(B)** Multilayer perceptron networks analysis. I: MLP networks, II: Confusion matrix, III: The model predicts 100% accuracy.

Yunnan province red soil (Figure 6): Compared with Group A, various microbial additives used in this experiment can cement the red soil and make the soil firm. In group C, calcium lignosulphonate (6 g/L, 8 g/L, 10 g/L) produced CaCO_3_ precipitation (marked with orange box), which made the soil surface whiter than other groups and calcium lignosulphonate 10 g/L CaCO_3_ precipitation was the most.

Henan province yellow soil (Figure 6): Compared with group A, the experimental group C calcium lignosulphonate (2 g/L, 4 g/L, 6 g/L, 8 g/L, 10 g/L), group D magnesium lignosulfonate (10 g/L), group E sodium lignin sulfonate (2 g/L), group F riboflavin (2 g/L, 6 g/L, 10 g/L) microbial additives can cement yellow soil and harden the soil.

Sichuan province purple soil (Figure 6): Compared with group A, various microbial additives in this experiment can cement purple soil and harden the soil.

Jiangsu province cyan soil (Figure 6): Compared with group A, various microbial additives in this experiment can cement cyan soil and harden the soil. Among them, group C calcium lignosulphonate (8 g/L, 10 g/L) produces CaCO_3_ precipitation (marked with orange box) so that the soil surface is whiter than other groups calcium lignosulphonate 10 g/L CaCO_3_ rainfall is the most.

Heilongjiang province black soil (Figure 6): Black soil is full of biological carbon, loose and porous, and rich in microbial content. Microbial additives have little effect on it.

### 3.9 SEM microscopic detection analysis

SEM-EDS was used for scanning cracks in concrete in this experiment further to observe the microstructure and elemental composition of concrete. Due to the complexity of soil structure and element content, it is unsuitable for experimental operations such as spraying gold and element detection. This experiment only analyzes the experimental group of concrete with a single element and smooth surface.

**Figure 7:**
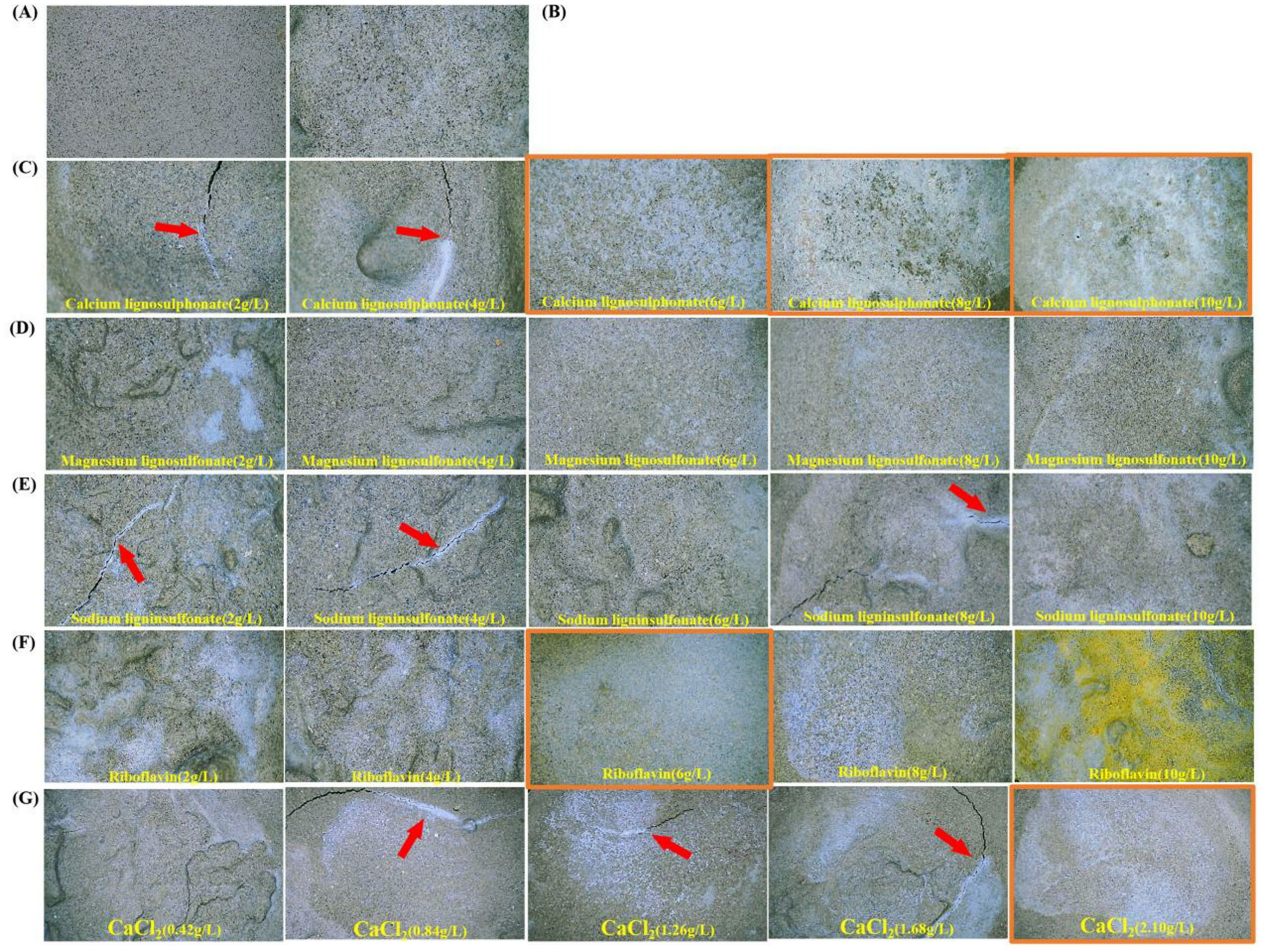
Stereomicroscopic images of concrete samples after 28 days of MICP treatment. **(A)** Blank Group A (untreated concrete). **(B)** Control Group B (concrete treated with *Bacillus subtilis* 168 only). **(C)** Group C treated with calcium lignosulfonate (2 g/L). **(D)** Group C treated with calcium lignosulfonate (4 g/L). **(E)** Group C treated with calcium lignosulfonate (6 g/L). **(F)** Group C treated with calcium lignosulfonate (8 g/L). **(G)** Group C treated with calcium lignosulfonate (10 g/L). Red arrows indicate cracks filled with CaCO₃ precipitates; orange boxes highlight areas of thick white CaCO₃ coverage.

Blank group A and control group B were set (Figure 7). As the Calcium lignosulphonate of group C (2 g/L, 4 g/L, 6 g/L, 8 g/L, 10 g/L) showed a good biological precipitation state under an industrial stereoscopic microscope 1,000×, SEM was used to conduct an in-depth state study on the microscopic state (Figure 7). The microscopic characterization of CaCO_3_ induced by *Bacillus subtilis* 168 microorganisms (high temperature and gold spraying environment show the bacteria in an apoptotic state, which does not affect the observation and data results). Because the soil contains natural CaCO_3_, such as marble and aragonite, an experimental error will be caused. This part only detects the experimental concrete with stable composition. The red arrow pointed rod-like *Bacillus subtilis* 168 (Group B, Group C calcium lignosulphonate (2 g/L, 4 g/L, 6 g/L, 8 g/L, 10 g/L) The diagram contains a large number of *Bacillus subtilis* 168. The arrows are only partially described). Yellow arrows refer to calcite crystallites (Group B, Group C calcium lignosulphonate (2 g/L, 4 g/L, 6 g/L, 8 g/L, 10 g/L) The diagram contains a large number of calcite crystallites. The arrows are only partially described).

The microstructure of calcium lignosulphonate (2 g/L, 4 g/L, 6 g/L, 8 g/L, 10 g/L) in groups A, B, and C was observed, and the white area of concrete under 1,000× magnification of industrial stereo electron microscope was mainly observed. SEM regulation was EHT = 5.00 kV, WD = 6.0 mm/8.5 mm. Signal A = InLens/SE2, Mag = 200X/500X/1.00KX/5.00KX/10.00KX/20.00KX/50.00KX, size = 50 μm, 20 μm, 10 μm, 2 μm, 1 μm, 500 nm, 200 nm. The occasional cracks in the calcium lignosulphonate (6 g/L, 10 g/L) of group C were filled with CaCO_3_ induced by many *Bacillus subtilis* 168, which was reflected in the above three-dimensional surface mirror and verified in the following XRD characterization. The group C calcium lignosulphonate (10 g/L) has rhombic calcite crystallites, aggregates of smaller crystals, confirming calcite morphology (Figure 7F). Similar SEM results can be found in other kinds of literature on *Bacillus subtilis* MICP (Ferral-Pérez et al., 2020; Hoffmann et al., 2021). Calcite is a stable form of calcium carbonate.

### 3.10 EDS spectral element composition analysis

Because the soil contains natural CaCO_3_, such as marble and aragonite will lead to experimental errors, this experiment observes the elemental composition of concrete; the overlapping images of elements inferred the distribution of CaCO_3_, Ca_3_SiO_5_, and other structures.

**Figure 8:**
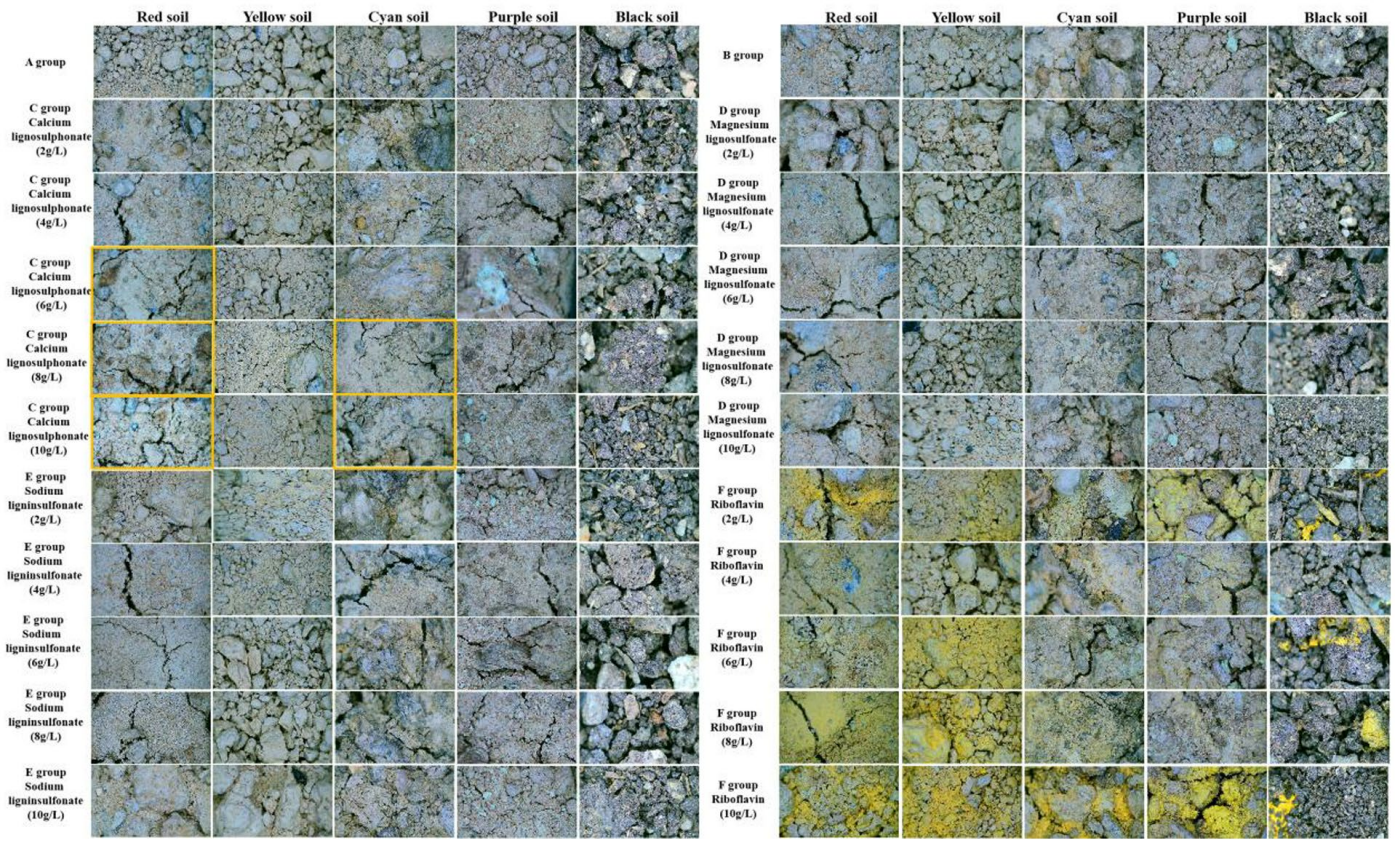
Stereomicroscopic images of different Chinese soil types after 28 days of MICP treatment. Rows represent treatment groups (**A**: blank; **B**: bacterial control; **C**: calcium lignosulfonate at 2, 4, 6, 8, 10 g/L; **D**: magnesium lignosulfonate; **E**: sodium lignosulfonate; **F**: riboflavin; **G**: CaCl₂), and columns represent soil types (red soil, yellow soil, cyan soil, purple soil, black soil). Orange boxes highlight areas with enhanced CaCO₃ precipitation and soil cementation.

Under SEM and stereomicroscope, it was observed that calcium lignosulphonate (10 g/L) in Group C had good biological precipitation MICP properties. To further investigate the characterization of bacterial additives at this concentration, this section detected the elemental composition of calcium lignosulphonate (10 g/L) crack areas in the experimental concrete with stable composition in Group C. As shown in Figure 8A, the results show that calcium lignosulphonate (10 g/L) in Group C exhibits significant Ca, C, and O peaks, and the precipitate is indeed CaCO_3_. Subsequent XRD characterization studies also confirmed this. Among the components produced by the synthesis of CaCO_3_ and Ca_3_SiO_5_ coprecipitation, the average content of C is 16.53% (peak area), the average content of O is 38.24%, the moderate content of Ca is 35.05%. The average content of Si is 2.57%. It was evident that the distribution of C, O, and Ca ions overlaps in the distribution map of each component, which indicates the distribution of CaCO_3_, most of which are cemented in the cracks, which can also confirm the results of MICP crack repair studied by a large number of teams (Supplementary Table S3 and Figure 8B).

### 3.11 XRD crystal characterization analysis

Granular precipitates on the surface of bacteria were observed in SEM-EDS detection. To further confirm whether it is CaCO_3_ crystal and which crystal form of CaCO_3_ crystal the precipitate is. From this, XRD was used to detect the particulate matter on the surface of crack bacteria. Group G (calcium source control experimental group) was added to this experiment to observe the microscopic characterization jointly to observe whether the Ca source will affect the crystal form. Because the soil containing natural CaCO_3_, such as marble, will lead to experimental error, this part only detects the experimental concrete with stable composition, due to the observation of good bio-precipitation MICP properties of Group C (calcium lignosulphonate) in stereomicroscope, SEM, and EDS detection. To delve deeper into the characterization of bacterial additives at this concentration, this experiment delves deeper into the relationship between calcium sources and crystallization. It adds the most common calcium source ion control group G (CaCl_2_) of MICP for comparative analysis. In this part, the experimental concrete blank group A, bacterial control group B, C group Calcium lignosulphonate, and calcium source control group G group (CaCl_2_) were detected.

**Figure 9:**
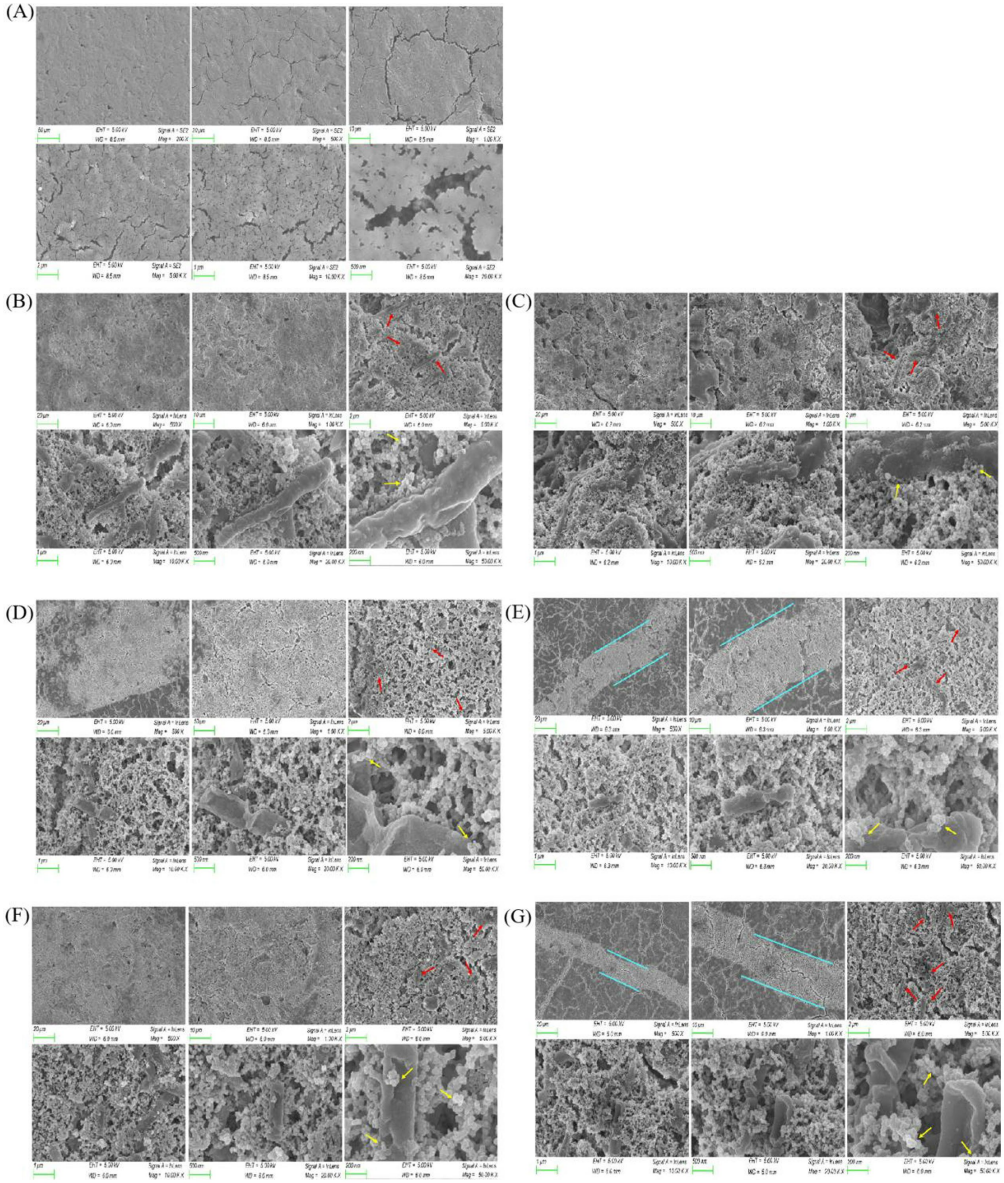
Sample diagram of *Bacillus subtilis* 168 detected by SEM. **(A)** SEM results of group A. **(B)** SEM results of group B. **(C)** SEM results of group C alcium lignosulphonate (2 g/L). **(D)** SEM results of group C calcium lignosulphonate (4 g/L). **(E)** SEM results of group C calcium lignosulphonate (6 g/L). **(F)** SEM results of group C calcium lignosulphonate (8 g/L). **(G)** SEM results of group C calcium lignosulphonate (10 g/L).

The XRD system comes with Jade software to identify the data of Group A, group B, group C (calcium lignosulphonate: 2 g/L, 4 g/L, 6 g/L, 8 g/L, 10 g/L), group G CaCl_2_ (0.42 g/L, 0.84 g/L, 1.26 g/L, 1.68 g/L, 2.10 g/L). Origin2022 software drawing. Surprisingly, it was found that the 18, 28, 34, 47, 50.5, and 54.5 peaks of Ca(OH)_2_ gradually decreased from group A to group C (calcium lignosulphonate: 10 g/L) until they disappeared. At the same time, the peak value of CaCO_3_ gradually increased, which indicated that CaCO_3_ gradually increased, corresponding to FT-IR characterization, appearance characterization, SEM characterization, and EDS characterization. Ca(OH)_2_ combines with CO_2_ metabolized by bacteria to generate CaCO_3_, which conforms to the traditional MICP formula and the concept of bacterial-induced biological precipitation (Figure 9A).

The peak of CaCO_3_ in Group G CaCl_2_ was similar. It was found that the 18 peaks of Ca(OH)_2_ gradually decreased from a concentration of Group G CaCl_2_(0.42 g/L) to Group G CaCl_2_(2.10 g/L) until they disappeared. The Ca(OH)_2_ 18 peaks of Group G CaCl_2_(1.68 g/L) and CaCl_2_(2.10 g/L) completely disappeared. As shown in Figure 9B, the equation is as follows:


$$\text{Cell}+{\mathrm{Ca}}^{2+}\to \;\;\;\;\text{Cell}\sim {\mathrm{Ca}}^{2+}$$ (1)

$${\mathrm{CO}}_{2}+H_{2}O\;\to \;\;{\mathrm{CO}}_{3}^{2-}+2H^{+}$$ (2)

$$\text{Cell}\sim {\mathrm{Ca}}^{2+}+{\mathrm{CO}}_{3}^{2-}\;\to \;\;\text{Cell}\sim {\text{CaCO}}_{3}$$ (3)

$$\mathrm{Ca}{\left(\mathrm{OH}\right)}_{2}+{\mathrm{CO}}_{2}\;\to \;\;{\text{CaCO}}_{3}+H_{2}O$$ (4)

$$\text{Cell}+C_{20}H_{24}{\mathrm{CaO}}_{10}S_{2}\to {\mathrm{CO}}_{2}+\text{Metabolite}+\text{Cell}\sim {\mathrm{Ca}}^{2+}$$ (5)

The reaction mechanisms of calcite formation in the proposed MICP system are presented in Equations (1)–(5). The difference in the intensity of calcium carbonate observed between different peaks in XRD may indicate that the calcite crystallites grow in a preferred orientation. The CaCO_3_ crystal forms of groups A, B, C, calcium lignosulphonate 10 g/L, and group G CaCl_2_ 2.10 g/L are compared (Figure 9C).

The A group 26.5° (101) Hexagon, A group 29° (211) Three parties (Rhombus). B group 26.5° (31–2) Monoclinic, B group 29° (104) Three parties (Hexagon). C calcium lignosulphonate 10 g/L group 26.5° (113) Hexagon, C calcium lignosulphonate 10 g/L group 29° (104) Three parties (Hexagon). G group CaCl_2_ 2.10 g/L group 26.5° (111) Orthogonal (Orthorhombic), G group CaCl_2_ 2.10 g/L group 29° (104) Hexagon. They are all shown as mixed crystal systems, which shows that the bacterial additives in this project have changed the CaCO_3_ crystal system in concrete.

Since the crystal form and surface properties determine its properties, physical properties such as crystal system change and stability have also been changed (Mann et al., 1991). It is known that the physical properties of calcite are the most stable at room temperature (Gorna et al., 2008). The porosity of MICP-precipitated calcite is less, indicating the close combination between calcite and other concrete components during the crack repair. The decrease in permeability is the result of higher density and reduced porosity. Calcite precipitation leads to a reduction in the permeability of biological concrete and improves durability. In addition, it has higher density and higher compressive strength results (Brugman et al., 2020). The crystal plane of calcite is 29° (104). Three parts (Hexagon). Group A, B, and C (calcium lignosulphonate 10 g/L) have a trigonal crystal system. At the same time, group C (calcium lignosulphonate 10 g/L) 29° (104) Three parts (Hexagon) is thinner, sharper, and narrower than other crystal systems, indicating that the reaction product is mainly calcite crystal. In addition, SEM characterization and surface characterization showed that most of the calcite crystallized in the cracks, so using group C (calcium lignosulphonate 10 g/L) additives was better than other groups. Aragonite belongs to the orthorhombic system G group (CaCl_2_ 2.10 g/L) 26.5° (111) orthorhombic. The structure’s calcium ions and carbonate ions are arranged in the most densely packed hexagonal manner. The structure is dense, a common state of natural precipitation (Ferral-Pérez et al., 2020).

### 3.12 FT-IR characterization analysis

**Figure 10:**
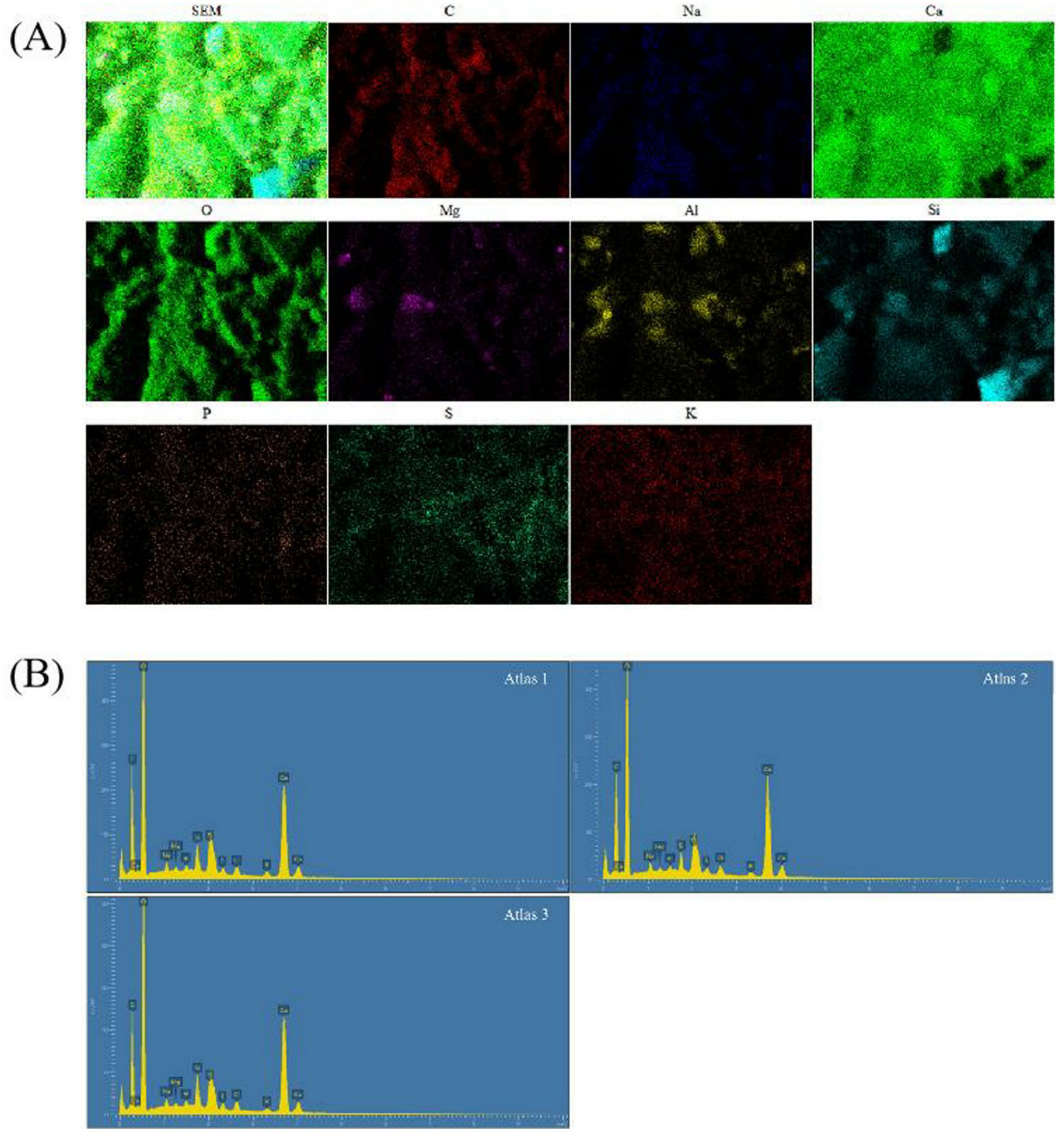
Sample diagram of *Bacillus subtilis* 168 detected by SEM and EDS. **(A)** EDS elements-distribution map results of group C calcium lignosulphonate (10 g/L). **(B)** EDS results of group C calcium lignosulphonate (10 g/L).

Due to the excellent Bio-precipitated calcium carbonate characterization of Group C under a stereomicroscope. This test compared the red soil of Yunnan Province, the yellow soil of Henan Province, the purple soil of Sichuan Province, the cyan soil of Jiangsu Province, and the black soil of Heilongjiang Province with the red soil, yellow soil, purple soil, cyan soil, and black soil added with C group (calcium lignosulphonate: 2 g/L, 4 g/L, 6 g/L, 8 g/L, 10 g/L). The absorption peak at approximately 1,487 cm^−1^ is an asymmetric stretching vibration peak with O=C=O, the characteristic peak of ${\mathrm{CO}}_{3}^{2-}$ in calcium carbonate. The characteristic peaks at about 875 cm^−1^ and 711 cm^−1^ are calcite characteristic absorption peaks (Figure 10). The O=C=O absorption peak at about 1,487 cm^−1^ in the yellow soil of Henan Province, the cyan soil of Jiangsu Province, and the black soil of Heilongjiang Province. The peak is gradually from the control soil to the C group (calcium lignosulphonate: 2 g/L, 4 g/L, 6 g/L, 8 g/L, 10 g/L), indicating that the content of CaCO_3_ is gradually increasing, which corresponds to the results of stereo microscope observation. The characteristic absorption peak of calcite in Yunnan red soil at about 875 cm^−1^, from the control blank red soil to group C (calcium lignosulphonate: 2 g/L, 4 g/L, 6 g/L, 8 g/L, 10 g/L) red soil; the peak is gradually apparent, indicating that the content of calcite is slowly increasing, which corresponds to the results of stereomicroscope observation.

### 3.13 Concrete samples

The single peak 3,643 cm^−1^ is the hydroxide radical peak of Ca(OH)_2_, in which group C (calcium lignosulphonate 8 g/L) and group C (calcium lignosulphonate 10 g/L) have no hydroxide radical stretching vibration peak, indicating that Ca(OH)_2_ has disappeared and transformed into calcite, echoing the XRD characterization test results. From group A, group B to group C, the hydroxide peak gradually weakened; in group C and group G, the hydroxide radical peak gradually weakened with the increase of calcium ions (Figure 10A).

In groups A, B, C, and G (CaCl_2_), the absorption peak at approximately 1,487 cm^−1^ is an asymmetric stretching vibration peak with O=C=O, indicating that this is the characteristic peak of ${\mathrm{CO}}_{3}^{2-}$ in calcium carbonate. The characteristic peaks at about 875 cm^−1^ and 711 cm^−1^ are calcite characteristic absorption peaks (Figure 10B).

The 857 cm^−1^ peak of group C (calcium lignosulphonate 10 g/L) is the smallest and sharpest, indicating that calcite is rich. And in Group C, the 857 cm^−1^ peak gradually became sharp with increasing calcium ion concentration. In contrast, Group G gradually became flat with increasing calcium ion concentration. This indicates that calcium lignosulphonate is beneficial for bacterial MICP biological precipitation with increasing concentration (Figure 10).

### 3.14 ATR-FTIR soil samples

Because group C has excellent characteristics of biological precipitation of calcium carbonate under a stereoscopic microscope. This test compared the red soil of Yunnan Province, the yellow soil of Henan Province, the purple soil of Sichuan Province, the cyan soil of Jiangsu Province, and the black soil of Heilongjiang Province with the red soil, yellow soil, purple soil, cyan soil, and black soil added with C group (calcium lignosulphonate: 2 g/L, 4 g/L, 6 g/L, 8 g/L, 10 g/L) (Figures 9C–G). The O=C=O absorption peak at about 1,487 cm^−1^ in the yellow soil of Henan Province, the cyan soil of Jiangsu Province, and the black soil of Heilongjiang Province. The peak is gradually from the control soil to the C group (calcium lignosulphonate: 2 g/L, 4 g/L, 6 g/L, 8 g/L, 10 g/L), indicating that the content of CaCO_3_ is gradually increasing, which corresponds to the results of stereo microscope observation. The characteristic absorption peak of calcite in Yunnan red soil at about 875 cm^−1^, from the control blank red soil to group C (calcium lignosulphonate: 2 g/L, 4 g/L, 6 g/L, 8 g/L, 10 g/L) red soil; the peak is gradually apparent, indicating that the content of calcite is slowly increasing, which corresponds to the results of stereomicroscope observation.

### 3.15 Raman and Raman microscopy analysis

To further confirm the accuracy of XRD in identifying crystal forms, Raman spectroscopy was used to detect the crystal forms of CaCO_3_ in the repetitive experimental group. Due to the observation of good bio-precipitation MICP properties in calcium lignosulphonate concrete under stereoscopic microscopy, SEM, EDS, XRD, FT-IR, and ATR detection, to further investigate the characterization of bacterial additives at this concentration, Raman was used to detecting Group A (blank group), Group B (control group), and Group C (detection group). Due to the high demand for Raman samples, this experiment repeated the experiments in Groups A, B, and C using curved glass dishes (Diameter 200 mm, height 20 mm).

**Figure 11:**
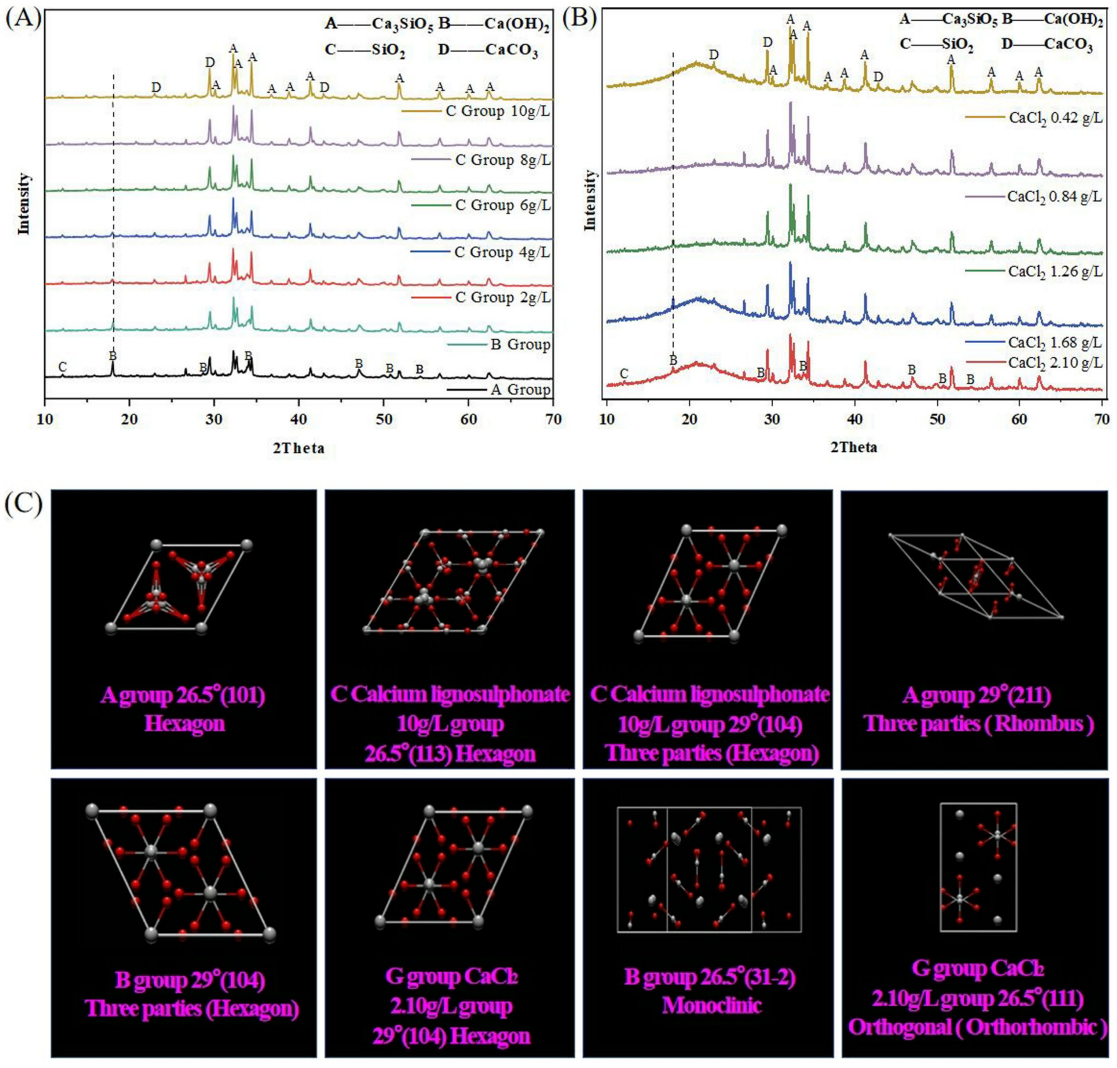
Sample diagram of *Bacillus subtilis* 168 detected by XRD. **(A)** XRD results for group A, group B and group C. **(B)** XRD results for group G. **(C)** XRD results for crystal resolution.

The Raman results are shown in Figure 11A: Group A has peaks of 151 cm^−1^, 280 cm^−1^, and 1,084 cm^−1^. The peaks of Group B were 152 cm^−1^, 280 cm^−1^, and 1,084 cm^−1^. The peak values of Group C (calcium lignosulphonate: 2 g/L) were 154 cm^−1^, 277 cm^−1^, and 1,084 cm^−1^. The peak values of Group C (calcium lignosulphonate: 4 g/L) were 154 cm^−1^, 277 cm^−1^, and 1,084 cm^−1^. The peak values of Group C (calcium lignosulphonate: 6 g/L) were 155 cm^−1^, 277 cm^−1^, and 1,084 cm^−1^. The peak values of Group C (calcium lignosulphonate: 8 g/L) were 152 cm^−1^, 281 cm^−1^, and 1,084 cm^−1^. The peak values of Group C (calcium lignosulphonate: 10 g/L) were 154 cm^−1^, 277 cm^−1^, 281 cm^−1^, and 1,084 cm^−1^. Wherein 154 cm^−1^ is calcite: the external translation lattice mode is S strong peak; 281 cm-1 is calcite: external free/rotating lattice mode is S strong peak (Parker et al., 2010); the most prominent S peak appears at 1,084 cm^−1^, which is caused by the symmetric stretching vibration of carbonate ions in minerals (Ševčík and Mácová, 2018). In addition, calcite belongs to the trigonal system, and carbonate ions present an equilateral triangle structure, which makes calcite show a stable mineral structure. However, aragonite is prone to transformation due to its rhombic crystal structure. Different crystal structures create other ion oscillation states, leading to various Raman spectral forms. In group C (calcium lignosulphonate: 8 g/L), there were two peaks at the same end, 277 cm^−1^ and 281 cm^−1^. It was considered that more than two kinds of CaCO_3_ crystal structures were mixed, indicating the existence of aragonite.

The peak shape of group C (calcium lignosulphonate 10 g/L) was consistent with calcite, the peak was narrow, and the peak was high, consistent with the results of stereomicroscope, SEM, EDS, XRD, FT-IR, and ATR.

### 3.16 Raman-microscopy results are shown below

The sample size of the repeated group is large and suitable for microscopic observation. In this test, Raman matching optical microscope was used to observe the sample of a curved glass dish (diameter 200 mm, height 20 mm). Compared with the blank sample of group A, a small amount of white film-like crystals (orange arrow label) were found in group B. Group C (calcium lignosulphonate 2 g/L) appeared with a small amount of CaCO_3_ crystal block (orange arrows marked). In group C (calcium lignosulphonate 4 g/L), many CaCO_3_ crystal blocks (orange arrow label) appeared. The crystallization of group C (Calcium lignosulphonate: 6 g/L, 8 g/L, 10 g/L) has been covered on the surface of concrete (Figure 11B).

## 4 Discussion

In this experiment, MICP and lignosulfonate-related information were mined through previous literature and computer simulation. The samples were detected by VITEK^®^MS, optical microscope, industrial stereo microscope, SEM, EDS, XRD, FTIR, Raman, and other detection technologies. Through the existing research theory, the metabolic process of *Bacillus subtilis* 168 to lignosulfonate was deeply explored. MICP bioinduced calcium carbonate precipitation from *Bacillus subtilis* group C (calcium lignosulfonate 8 g/L) and group C (calcium lignosulfonate 10 g/L) was applied to concrete and soil in China (Yunnan laterite, Henan yellow soil, Sichuan purple soil, Jiangsu cyan soil, Heilongjiang black soil). Group C (calcium lignosulphonate 10 g/L) MICP biologically induced calcium carbonate precipitation can make Chinese soil cementation better, compared with other groups A, B, D, E, F, and G in the experiment; group C (calcium lignosulphonate 10 g/L) can produce a large number of structurally stable CaCO_3_ (calcite) crystals. At the same time, it was found that the concentration of group C (calcium lignosulphonate 8 g/L) and group C (calcium lignosulphonate 10 g/L) could transform Ca(OH)_2_ into CaCO_3_ in concrete, which explained a new way of MICP biologically induced calcium carbonate precipitation. *Bacillus subtilis* 168 demonstrates strong alkali resistance, thriving in highly alkaline environments like concrete, where its metabolic enzymes, such as lipase and laccase, show peak activity at pH levels of 10.0 and 9.0, respectively, enhancing the degradation of lignin and its derivatives (Lesuisse et al., 1993; Wang et al., 2010; Wilks et al., 2009). In a computer simulation, the BsDyP enzyme fits well with the lignosulfonate parent molecule. Heterologous expression of genes from *Bacillus subtilis* can drive MICP growth, with studies showing that *Bacillus subtilis* 168 treatment increases the compressive strength of cement mortar by approximately 19.5%, although the surrounding chemical properties significantly influence the rate of calcite production and its distribution (Hoffmann et al., 2021; Park et al., 2012; Rana et al., 2023). The optimal binding energy is −8.8 kcal/mol, indicating that it has a great interaction force. In the ssu series of enzymes, ssuD also has a great force with the lignosulfonate parent molecule. Other team studies have confirmed that ssuD is a protease that accepts sulfonates. Whether nutrients pollute the environment, the number of CaCO_3_ crystals, the type of CaCO_3_ crystals, and whether the applied strains are pathogenic bacteria, our MICP scheme has advantages and is suitable for environmental applications. These multi-method results provide substantial evidence that calcium lignosulfonate enhances MICP mineralization, though they do not constitute a final validation of its engineering performance.

**Figure 12:**
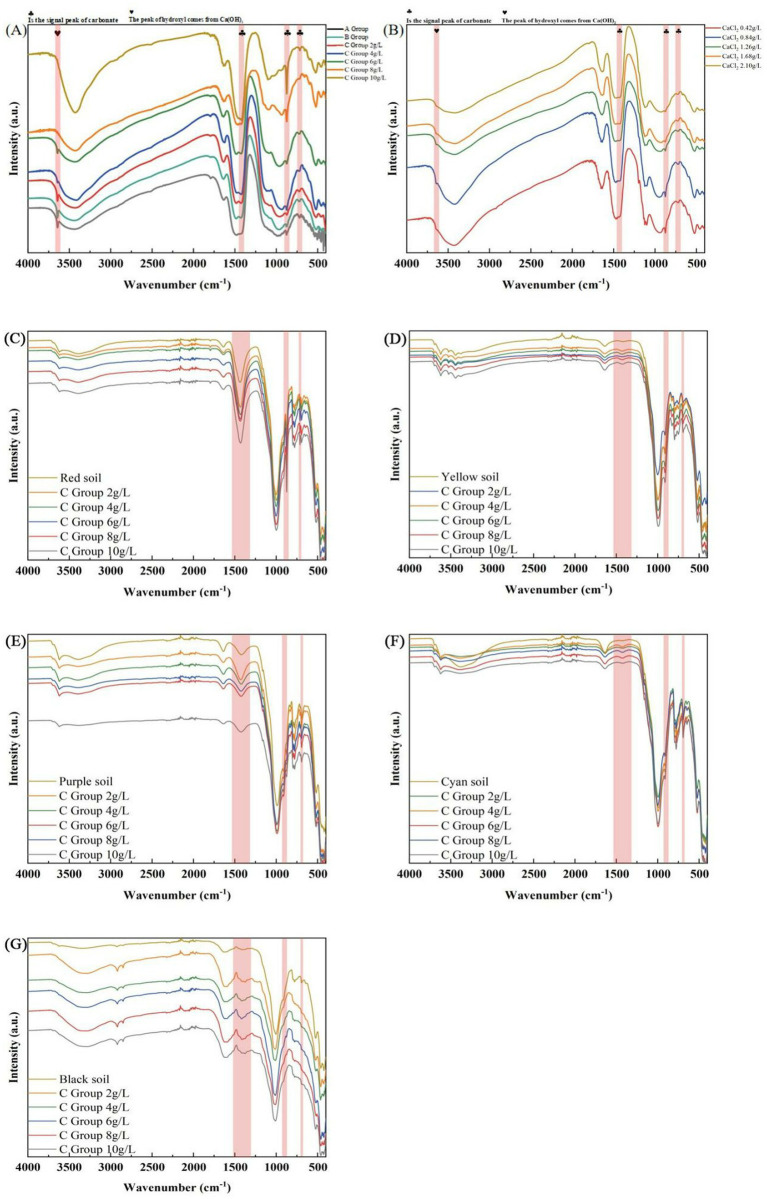
Sample diagram of *Bacillus subtilis* 168 detected by FT-IR. **(A)** FT-IR results for group A, group B and group C. **(B)** FT-IR results for group G. **(C)** FT-IR results for red soil. **(D)** FT-IR results for yellow soil. **(E)** FT-IR results for purple soil. **(F)** FT-IR results for cyan soil. **(G)** FT-IR results for black soil.

**Figure 13:**
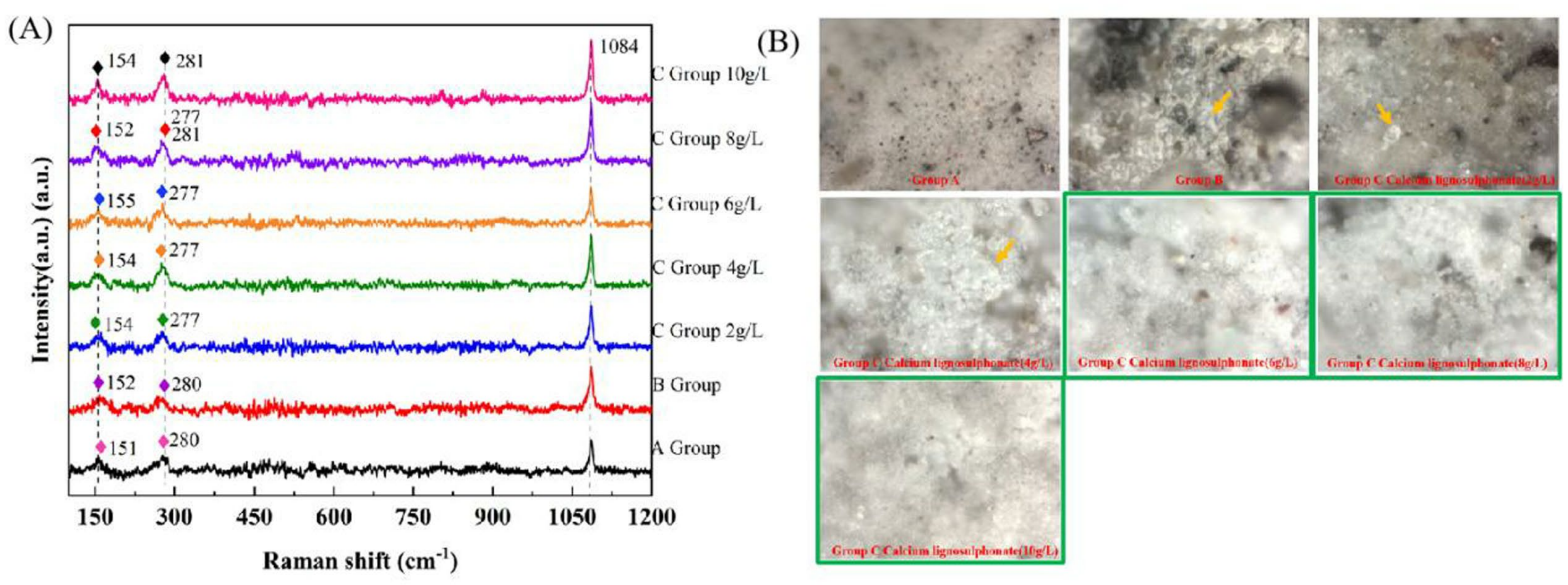
Sample diagram of *Bacillus subtilis* 168 detected by Raman. **(A)** Raman results for group A, group B and group C. **(B)** Raman-microscopy results for group A, group B and group C.

**Figure 14:**
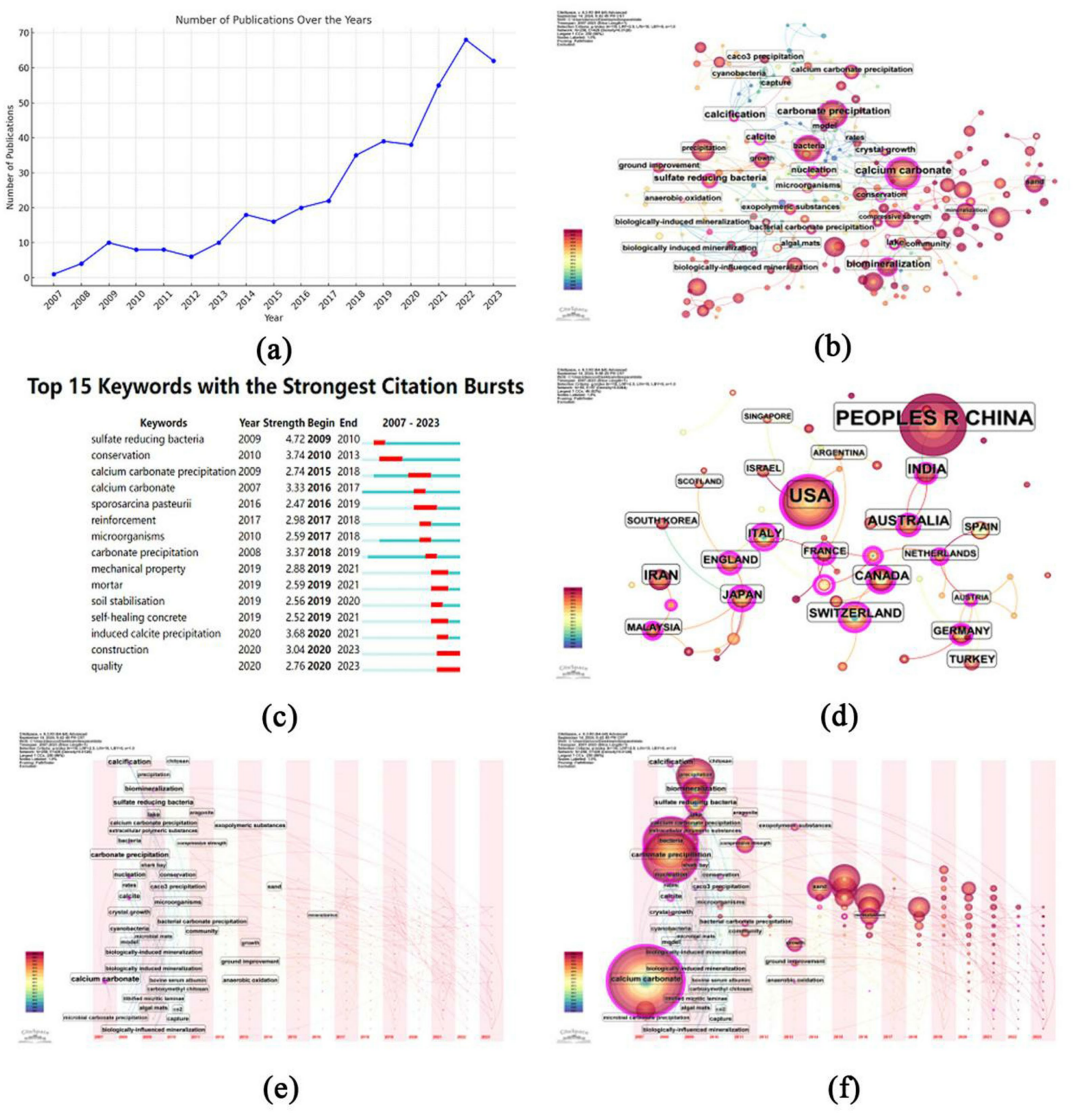
Literature search results. **(a)** Annual growth of articles since 2007. **(b)** Co-occurrence of countries involved in MICP research. **(c)** Keyword co-occurrence mapping. **(d)** Time zone map. **(e)** Keyword burst analysis. **(f)** Visual mapping of MICP evolution.

Based on the bibliometric analysis of Web of Science data, in recent years, microbial-induced calcium carbonate precipitation (MICP) has gained significant attention, particularly as an eco-friendly solution for soil remediation and concrete stabilization. The bibliometric analysis using data from Web of Science reflects the increasing research interest in this field. As depicted in Figure 12A, the number of publications on MICP has shown a sharp upward trend since 2007, with a substantial rise after 2016, reflecting the growing academic and industrial interest in developing environmentally sustainable methods for soil and concrete repair (Fouladi et al., 2023; Jiang et al., 2023; Wang et al., 2023). The publication count peaks in 2022, indicating that MICP is currently a hot research area. The co-occurrence of countries involved in MICP research, shown in Figure 12B, shows that China and the United States dominate the field, with significant contributions from countries like India, Australia, and Canada. This suggests that both developed and developing nations are heavily invested in the exploration and application of MICP, likely due to its potential to address global environmental challenges such as soil erosion and the sustainability of concrete infrastructure. Furthermore, the collaboration networks between these nations suggest that MICP is a highly interdisciplinary and internationally collaborative field. Keyword co-occurrence mapping (Figure 12C) indicates the central themes in the field, with “calcium carbonate,” “carbonate precipitation,” “bacteria,” and “calcification” being the most frequently occurring terms. This reinforces the fundamental role that bacteria such as *Bacillus subtilis* play in calcium carbonate precipitation and their applications in various contexts, including soil remediation and construction. Moreover, key terms like “biomineralization” and “sulfate reducing bacteria” underscore the diverse metabolic pathways and microbial systems explored for optimizing MICP processes. The time zone map presented in Figure 12D highlights the chronological development of keywords associated with MICP research. Earlier studies focused on general concepts such as “calcium carbonate” and “precipitation.” However, as the field has progressed, more specialized terms like “biologically induced mineralization” and “self-healing concrete” have emerged, indicating a shift toward applied research and the refinement of MICP techniques for practical use in construction and environmental management. Further supporting this shift is the keyword burst analysis in Figure 12E, which identifies the keywords with the strongest citation bursts over the years. The prominence of terms like “calcium carbonate precipitation,” “microorganisms,” and “reinforcement” from 2017 onwards shows the field’s movement toward applied microbial systems for enhancing material properties, particularly in the context of concrete and soil stability. The visual mapping of the field’s evolution in Figures 12F,G clearly illustrates how research themes have expanded and become more specialized over time. Early research focused primarily on fundamental processes like “carbonate precipitation” and “calcification,” while recent studies delve into niche applications like “self-healing concrete” and “soil stabilization.” This development trend highlights the growing recognition of MICP’s potential for sustainable construction practices and environmental conservation (see Figures 13, 14).

As early as 15 years ago, studies suggested that oral administration of *Bacillus subtilis* should be safe and supported using *Bacillus subtilis* as a food supplement (Hong et al., 2008). Moreover, *Bacillus subtilis* fermented natto can be a healthy food (Ruiz Sella et al., 2021).

However, most of the traditional MICP bacteria are pathogenic bacteria, such as *Bacillus cereus* (common bacteria causing food poisoning) (Granum and Lindbäck, 2012), *Streptococcus pasteurii* (inducing sepsis) (Alex et al., 2013; Cuthbert et al., 2012), *Salmonella pasteurii* (inducing food poisoning), drinking water-borne pathogens (Louboudy et al., 2007; Tang et al., 2020), *Pseudomonas aeruginosa* (the most common drug-resistant bacteria in nosocomial infections) (Wath and Pusadkar, 2020; Wu et al., 2015), *Bacillus sphaericus*, *Bacillus cohnii*, and detoxifying bacteria (Cheng et al., 2013). A detailed comparison between the traditional MICP process and our MICP process is provided in Supplementary Table S4. The MICP safety of *Bacillus subtilis* is higher than that of other bacteria. At the same time, *Bacillus subtilis* inhibits harmful bacteria (Hashem et al., 2019), playing the same role as chemical fertilizers and insecticides as biological fertilizers and biological insecticides. Plant growth-promoting rhizobacteria (PGPR) can significantly promote plant growth and represent plant-microorganism interactions. It has been reported that some teams have used *Bacillus subtilis* as the preferred bacteria for MICP (Sharma et al., 2021) for sand stabilization. The GEO (Gene Expression Omnibus) study of *Bacillus subtilis* described the coordinated formation of calcite scaffold to support the morphogenesis of the biofilm of *Bacillus subtilis*. The research results confirmed the process necessary for the development of the biofilm of *Bacillus subtilis*—specialized cells strictly regulated the formation of the mineral scaffold and proved that *Bacillus subtilis* generate calcite from the perspective of genetics, and the biofilm morphology is calcium-dependent (Keren-Paz et al., 2022).

There are many experiments and patents to obtain adhesives that enhance the adhesion of wood chips from sulfate lignin or lignosulfonate treated with *Bacillus subtilis* laccase (Widsten and Kandelbauer, 2008). For the restructuring of cellulose to make it more adhesive. Studies have shown that Ca-lignin sulfonate is matched with vanillic acid (*m*/*z* 169 MH, 191 MNa) by enzymatic hydrolysis compounds (Kaczmarek et al., 2017). The metabolites of lignosulfonate were analyzed, KEGG results showed that the sulfonates were oxidized by redox reaction → alkane sulfonate → sulfite → sulfate.

The research shows that the protein of the best docking affinity of lignosulfonate molecules in *Bacillus subtilis* 168 is BsDyP. Among heme peroxidase enzymes, DyP-type peroxidase belongs to a new superfamily of heme peroxidase enzymes, which have stability under acidic conditions and potential applications in various dye degradation and lignin degradation (Dhankhar et al., 2020). BsDyP was identified as the first bacterial peroxidase capable of oxidizing high redox potential lignin-related model compounds (especially VGE), revealing the previously unknown versatility of lignin degradation biocatalysts in nature (Min et al., 2015). BsDyP decomposes dimeric lignin VGE by cutting C at 50 °C. Studies have shown that the crystal structure of the BsDyP-veratryl alcohol (VA) complex has deeply studied the binding of small substrate molecules in the DyP heme cavity. The turnover number of VA oxidized by BsDyP was 0.065 s^−1^, and then 2, 6-dimethoxyphenol (DMP) and guaiacol were oxidized.

In the traditional MICP equation: the presence of by-product ammonia, excessive ammonia will accelerate eutrophication, and depletion of dissolved oxygen in the environment (Chen et al., 2019).


$$\begin{array}{l} \mathrm{CO}{\left({\mathrm{NH}}_{2}\right)}_{2}+2H_{2}O\;\to \;2{\mathrm{NH}}^{4+}+\\ {\mathrm{CO}}_{3}^{2-}(\text{in presence of urease from bacteria}) \end{array}$$ (6)

$${\text{CaCl}}_{2}+H_{2}O\to {\mathrm{Ca}}^{2+}+2{\mathrm{Cl}}^{-}$$ (7)

$${\mathrm{Ca}}^{2+}+{\mathrm{CO}}_{3}^{2-}\to {\text{CaCO}}_{3}↓$$ (8)

The reaction principle of conventional urea-based MICP is shown in Equations (6)–(8). However, compared with the traditional MICP, the advantage of this design lies in this experiment altogether avoided ammonia production, and lignosulfonate decomposition was achieved. At the same time, calcium lignosulfonate is a very excellent concrete water-reducing agent and soil stabilizer. The safety problem can be guaranteed, and the concentration added in this experiment meets the national content standard of China (Grierson et al., 2005). This study proposes a new metabolic pathway: Ca (OH)_2_ is converted into CaCO_3_, which can be reflected in XRD, FT-IR and Raman characterization.

This study shows that MICP can be used to repair cracks in building engineering. Concrete fracture is not conducive to structural performance in terms of service life. Applying MICP of bacteria to repair concrete cracks to overcome; MLCP has been used to repair concrete cracks, reduce the porosity of concrete through pore blockage, treat the surface of building materials, produce Bio-cement for sandstone bricks, induce concrete surface coatings, and stably disperse soil (Iqbal et al., 2021). A critical review of MICP applications can deepen the understanding of the role of Bio-cementing in sustainable building construction (Mutitu et al., 2019). Evaluating the mechanical and chemical properties of Bio-cemented building materials, their robustness and durability can be characterized.

There are still some shortcomings in this experiment, and we will carry out follow-up research. Many MICP reports worldwide contain MICP concrete 28 days culture compression, tensile, fracture resistance, frost resistance, impermeability, and other experiments. This experiment focuses on the formulation and concentration ratio of additives. In the future, under the professional guidance of civil engineering experts, the team will follow up the experiments on soil landslide, sand resistance, concrete compression resistance, tensile resistance, fracture resistance, frost resistance, and impermeability. It has been found that *Bacillus subtilis* 168 has radiation resistance (Wassmann et al., 2010) and has been applied in space environments and simulated Mars environments (Morrison et al., 2019; Wassmann et al., 2012). This experimental concrete additive formula is used to the space environment MICP acts on the space station, and even the transformation of soil and repair of concrete in other space environments is the team’s goal and expectation (Horneck, 1993). Furthermore, the lignin degradation process facilitated by *Bacillus subtilis* produces minimal environmental contaminants, improving overall eco-friendliness. The observed mineralization-promoting effect of calcium lignosulfonate provides a theoretical basis for its scaled-up application, and further work is needed to validate its performance in large-scale soil stabilization and infrastructure repair projects, especially in regions with severe environmental degradation.

## 5 Conclusion

These results confirm the feasibility of using calcium lignosulfonate as an eco-friendly alternative energy source in MICP, as well as its ability to enhance mineralization and its potential for concrete restoration and soil remediation.

- The experimental results indicate that *Bacillus subtilis* 168 can metabolize calcium lignosulfonate as a carbon source, enhancing calcite formation with external calcium supplied by CaCl₂.
- Compared to traditional urea-based MICP systems, the use of calcium lignosulfonate significantly reduces ammonia emissions and environmental impact.
- The study showed that calcium lignosulfonate at 10 g/L produced the highest amount of calcite in various soil types, including Yunnan red soil and Henan yellow soil, effectively cementing the soil. In concrete samples, the formation of stable CaCO_3_ crystals was observed, which improved the mechanical properties of the concrete and facilitated crack repair.
- Molecular docking analysis revealed that key enzymes like BsDyP and Ssu in *Bacillus subtilis* were involved in the lignosulfonate metabolism, further enhancing the MICP process.
- These findings suggest that calcium lignosulfonate can act as a sustainable and efficient alternative carbon and energy source to urea in MICP systems, offering a promising solution for environmental and infrastructural applications.

## Data Availability

The original contributions presented in the study are included in the article/Supplementary material, further inquiries can be directed to the corresponding authors.

## Footnotes

1
https://pubchem.ncbi.nlm.nih.gov

2
https://link.zhihu.com/?target=http%3A//zinc15.docking.org/

3
https://www.rcsb.org/

4
http://labshare.cn/clab/index.html
