# Supplementary material for: Probiotics use environmentally friendly calcium lignosulfonate as an energy source to MICP-acting on concrete and soil remediation
Source: Front Microbiol. 2026 May 11;17:1826075. doi: 10.3389/fmicb.2026.1826075 (PMC13199252; doi:10.3389/fmicb.2026.1826075)
Supplement: Supplementary file 1 [file Table_1.DOCX]

Table S1: Preparation formula of concrete (P.O42.5 ) and soil samples.

| **Groupin g of additives and serial number of samples** | | | | | | | | | | | | | | | | | | | | | | | | | | | | | | | | | | | | | | | | | | | | | | | | | | | | | | | | | | | | | | | | | | | | | | | | | | | | | | | | | | | | | | | | |
| --- | --- | --- | --- | --- | --- | --- | --- | --- | --- | --- | --- | --- | --- | --- | --- | --- | --- | --- | --- | --- | --- | --- | --- | --- | --- | --- | --- | --- | --- | --- | --- | --- | --- | --- | --- | --- | --- | --- | --- | --- | --- | --- | --- | --- | --- | --- | --- | --- | --- | --- | --- | --- | --- | --- | --- | --- | --- | --- | --- | --- | --- | --- | --- | --- | --- | --- | --- | --- | --- | --- | --- | --- | --- | --- | --- | --- | --- | --- | --- | --- | --- | --- | --- | --- | --- | --- | --- | --- |
| **Sample types** | | | | | | | | | | | | | | | | | | | **The A group** | | | | | | | | | | | | | | | | | | | | | | | | | | | | | | | | | | | | | | | | | | | | | | | | | | | | | | | | | | | | | | **The total amount of each component of the sample** | | | | | | | |
| Concrete | | | | | | | | | | | P.O42.5+H_2_O  Repeat 3 times (For stereo microscopy) | | | | | | | | | | | | | | | | | | | | P.O42.5+H_2_O  Repeat 3 times (For infrared spectroscopy) | | | | | | | | | | | | | | | | | | | | P.O42.5+H_2_O  Repeat 3 times (For powder XRD) | | | | | | | | | | | | | | | | | | | | | | P.O42.5+H_2_O  Repeat 3 times (For SEM, EDS) | | | | | | | | | | | | | | | P.O42.5 used 12 g  H_2_O used 3.84 mL |
| Yunnan Province Red soil | | | | | | | | | | | | | | | | | | | Red soil in Yunnan Province: repeat 3 times | | | | | | | | | | | | | | | | | | | | | | | | | | | | | | | | | | | | | | | | | | | | | | | | | | | | | | | | | | | | | | Red soil used 9 g | | | | | | | |
| Henan Province Yellow soil | | | | | | | | | | | | | | | | | | | Yellow soil in Henan Province: repeat 3 times | | | | | | | | | | | | | | | | | | | | | | | | | | | | | | | | | | | | | | | | | | | | | | | | | | | | | | | | | | | | | | Yellow soil used 9 g | | | | | | | |
| Sichuan Province Purple soil | | | | | | | | | | | | | | | | | | | Purple soil in Sichuan Province: repeat 3 times | | | | | | | | | | | | | | | | | | | | | | | | | | | | | | | | | | | | | | | | | | | | | | | | | | | | | | | | | | | | | | Purple soil used 9 g | | | | | | | |
| Jian gsu Province Cyan Soil | | | | | | | | | | | | | | | | | | | Cyan Soil in Jiangsu Province: repeat 3 times | | | | | | | | | | | | | | | | | | | | | | | | | | | | | | | | | | | | | | | | | | | | | | | | | | | | | | | | | | | | | | Cyan Soil used 9 g | | | | | | | |
| Heilon gjiang Province Black soil | | | | | | | | | | | | | | | | | | | Black soil in Heilongjian g Province: repeat 3 times | | | | | | | | | | | | | | | | | | | | | | | | | | | | | | | | | | | | | | | | | | | | | | | | | | | | | | | | | | | | | | Black soil used 9 g | | | | | | | |
| **Sample types** | | | | | | | | | | | | | | | | | | | **The B group** | | | | | | | | | | | | | | | | | | | | | | | | | | | | | | | | | | | | | | | | | | | | | | | | | | | | | | | | | | | | | | **The total amount of each component of the sample** | | | | | | | |
| Concrete | | | | | | | | | | P.O42.5+H_2_O+ Bacterial additive solution  Repeat 3 times | | | | | | | | | | | | | | | | | | | | P.O42.5+H_2_O+ Bacterial additive solution  Repeat 3 times | | | | | | | | | | | | | | | | | | | P.O42.5+H_2_O+ Bacterial additive solution  Repeat 3 times | | | | | | | | | | | | | | | | P.O42.5+H_2_O+ Bacterial additive solution  Repeat 3 times | | | | | | | | | | | | | | | | P.O42.5 used 12 g  H_2_O used 3.84 mL  Bacterial additive solution used 3.6 mL | | | | | | | |
| Yunnan Province Red soil | | | | | | | | | | | | | | | | Red soil in Yunnan Province + Bacterial additive solution: repeat 3 times | | | | | | | | | | | | | | | | | | | | | | | | | | | | | | | | | | | | | | | | | | | | | | | | | | | | | | | | | | | | | | | | | | | | | | | Red soil used 9 g  Bacterial additive solution used 0.9 mL | |
| Henan Province Yellow soil | | | | | | | | | | | | | | | | Yellow soil in Henan Province + Bacterial additive solution: repeat 3 times | | | | | | | | | | | | | | | | | | | | | | | | | | | | | | | | | | | | | | | | | | | | | | | | | | | | | | | | | | | | | | | | | | | | | | | Yellow soil used 9 g  Bacterial additive solution used 0.9 mL | |
| Sichuan Province Purple soil | | | | | | | | | | | | | | | | Purple soil in Sichuan Province + Bacterial additive solution: repeat 3 times | | | | | | | | | | | | | | | | | | | | | | | | | | | | | | | | | | | | | | | | | | | | | | | | | | | | | | | | | | | | | | | | | | | | | | | Purple soil used 9 g  Bacterial additive solution used 0.9 mL | |
| Jian gsu Province Cyan Soil | | | | | | | | | | | | | | | | Cyan Soil in Jian gsu Province + Bacterial additive solution: repeat 3 times | | | | | | | | | | | | | | | | | | | | | | | | | | | | | | | | | | | | | | | | | | | | | | | | | | | | | | | | | | | | | | | | | | | | | | | Cyan Soil used 9 g  Bacterial additive solution used 0.9 mL | |
| Heilon gjian g Province Black soil | | | | | | | | | | | | | | | | Black soil in Heilongjiang Province + Bacterial additive solution: repeat 3 times | | | | | | | | | | | | | | | | | | | | | | | | | | | | | | | | | | | | | | | | | | | | | | | | | | | | | | | | | | | | | | | | | | | | | | | Black soil used 9 g  Bacterial additive solution used 0.9 mL | |
| **Sample types** | | | | | | | **The C group Calcium lignosulphonate (2 g/L)** | | | | | | | | | | | | | | | | | | | | | | **The C group Calcium lignosulphonate (4 g/L)** | | | | | | | | | | | | | | | | | | **The C group Calcium lignosulphonate (6 g/L)** | | | | | | | | | | | | | | | | **The C group Calcium lignosulphonate (8 g/L)** | | | | | | | | | | | **The C group Calcium lignosulphonate (10 g/L)** | | | | | | | | | | | | | **The total amount of each component of the sample** | |
| Concrete | | P.O42.5 +Calcium lignosulphonate (2 g/L) + Bacterial additive solution: repeat 12 times | | | | | | | | | | P.O42.5 +Calcium lignosulphonate (4 g/L) + Bacterial additive solution: repeat 12 times | | | | | | | | | | | | | | | | | | | | P.O42.5 + Calcium lignosulphonate (6 g/L) + Bacterial additive solution: repeat 12 times | | | | | | | | | | | | | | | | P.O42.5+ Calcium lignosulphonate (8 g/L) + Bacterial additive solution: repeat 12 times | | | | | | | | | | | | | | | | P.O42.5 + Calcium lignosulphonate (10 g/L) + Bacterial additive solution: repeat 12 times | | | | | | | | | | P.O42.5 used 60 g  Calcium lignosulphonate (2 g/L) solution used 3.84 mL  Calcium lignosulphonate (4 g/L) solution used 3.84 mL  Calcium lignosulphonate (6 g/L) solution used 3.84 mL  Calcium lignosulphonate (8 g/L) solution used 3.84 mL  Calcium lignosulphonate (10 g/L) solution used 3.84 mL  Bacterial additive solution used 18 mL | | | | | | | | | | | | | | |
| Yunnan Province Red soil | | | | | | | Red soil in Yunnan Province +Calcium lignosulphonate (2 g/L) + Bacterial additive solution: repeat 3 times | | | | | | | | | | Red soil in Yunnan Province +Calcium lignosulphonate (4 g/L) + Bacterial additive solution: repeat 3 times | | | | | | | | | | | | | | | | | | | Red soil in Yunnan Province +Calcium lignosulphonate (6 g/L) + Bacterial additive solution: repeat 3 times | | | | | | | | | | | | | | | | | | Red soil in Yunnan Province +Calcium lignosulphonate (8 g/L) + Bacterial additive solution: repeat 3 times | | | | | | | | | | | | Red soil in Yunnan Province +Calcium lignosulphonate (10 g/L) + Bacterial additive solution: repeat 3 times | | | | | | | | | | | Red soil used 45 g  Calcium lignosulphonate (2 g/L) solution used 0.9 mL  Calcium lignosulphonate (4 g/L) solution used 0.9 mL  Calcium lignosulphonate (6 g/L) solution used 0.9 mL  Calcium lignosulphonate (8 g/L) solution used 0.9 mL  Calcium lignosulphonate (10 g/L) solution used 0.9 mL  Bacterial additive solution used 4.5 mL | | | | | | | | | | | |
| Henan Province Yellow soil | | | | | | | Yellow soil in Henan Province +Calcium lignosulphonate (2 g/L) + Bacterial additive solution: repeat 3 times | | | | | | | | | | Yellow soil in Henan Province +Calcium lignosulphonate (4 g/L) + Bacterial additive solution: repeat 3 times | | | | | | | | | | | | | | | | | | | Yellow soil in Henan Province +Calcium lignosulphonate (6 g/L) + Bacterial additive solution: repeat 3 times | | | | | | | | | | | | | | | | | | Yellow soil in Henan Province +Calcium lignosulphonate (8 g/L) + Bacterial additive solution: repeat 3 times | | | | | | | | | | | | Yellow soil in Henan Province +Calcium lignosulphonate (10 g/L) + Bacterial additive solution: repeat 3 times | | | | | | | | | | | Yellow soil used 45 g  Calcium lignosulphonate (2 g/L) solution used 0.9 mL  Calcium lignosulphonate (4 g/L) solution used 0.9 mL  Calcium lignosulphonate (6 g/L) solution used 0.9 mL  Calcium lignosulphonate (8 g/L) solution used 0.9 mL  Calcium lignosulphonate (10 g/L) solution used 0.9 mL  Bacterial additive solution used 4.5 mL | | | | | | | | | | | |
| Sichuan Province Purple soil | | | | | | | Purple soil in Sichuan Province +Calcium lignosulphonate (2 g/L) +Bacterial additive solution: repeat 3 times | | | | | | | | | | Purple soil in Sichuan Province +Calcium lignosulphonate (4 g/L) +Bacterial additive solution: repeat 3 times | | | | | | | | | | | | | | | | | | | Purple soil in Sichuan Province +Calcium lignosulphonate (6 g/L) +Bacterial additive solution: repeat 3 times | | | | | | | | | | | | | | | | | | Purple soil in Sichuan Province +Calcium lignosulphonate (8 g/L) +Bacterial additive solution: repeat 3 times | | | | | | | | | | | | Purple soil in Sichuan Province +Calcium lignosulphonate (10 g/L) +Bacterial additive solution: repeat 3 times | | | | | | | | | | | Purple soil used 45 g  Calcium lignosulphonate (2 g/L) solution used 0.9 mL  Calcium lignosulphonate (4 g/L) solution used 0.9 mL  Calcium lignosulphonate (6 g/L) solution used 0.9 mL  Calcium lignosulphonate (8 g/L) solution used 0.9 mL  Calcium lignosulphonate (10 g/L) solution used 0.9 mL  Bacterial additive solution used 4.5 mL | | | | | | | | | | | |
| Jian gsu Province  Cyan Soil | | | | | | | Cyan Soil in Jiangsu Province +Calcium lignosulphonate (2 g/L) +Bacterial additive solution: repeat 3 times | | | | | | | | | | Cyan Soil in Jiangsu Province +Calcium lignosulphonate (4 g/L) +Bacterial additive solution: repeat 3 times | | | | | | | | | | | | | | | | | | | Cyan Soil in Jiangsu Province +Calcium lignosulphonate (6 g/L) +Bacterial additive solution: repeat 3 times | | | | | | | | | | | | | | | | | | Cyan Soil in Jiangsu Province +Calcium lignosulphonate (8 g/L) +Bacterial additive solution: repeat 3 times | | | | | | | | | | | | Cyan Soil in Jiangsu Province +Calcium lignosulphonate (10 g/L) +Bacterial additive solution: repeat 3 times | | | | | | | | | | | Cyan Soil used 45 g  Calcium lignosulphonate (2 g/L) solution used 0.9 mL  Calcium lignosulphonate (4 g/L) solution used 0.9 mL  Calcium lignosulphonate (6 g/L) solution used 0.9 mL  Calcium lignosulphonate (8 g/L) solution used 0.9 mL  Calcium lignosulphonate (10 g/L) solution used 0.9 mL  Bacterial additive solution used 4.5 mL | | | | | | | | | | | |
| Heilon gjian g Province Black soil | | | | | | | Black soil in Heilon gjian g Province+Calcium lignosulphonate (2 g/L) +Bacterial additive solution: repeat 3 times | | | | | | | | | | Black soil in Heilon gjian g Province+Calcium lignosulphonate (4 g/L) +Bacterial additive solution: repeat 3 times | | | | | | | | | | | | | | | | | | | Black soil in Heilon gjian g Province+Calcium lignosulphonate (6 g/L) +Bacterial additive solution: repeat 3 times | | | | | | | | | | | | | | | | | | Black soil in Heilon gjian g Province+Calcium lignosulphonate (8 g/L) +Bacterial additive solution: repeat 3 times | | | | | | | | | | | | Black soil in Heilon gjian g Province+Calcium lignosulphonate (10 g/L) +Bacterial additive solution: repeat 3 times | | | | | | | | | | | | | Black soil used 45 g  Calcium lignosulphonate (2 g/L) solution used 0.9 mL  Calcium lignosulphonate (4 g/L) solution used 0.9 mL  Calcium lignosulphonate (6 g/L) solution used 0.9 mL  Calcium lignosulphonate (8 g/L) solution used 0.9 mL  Calcium lignosulphonate (10 g/L) solution used 0.9 mL  Bacterial additive solution used 4.5 mL | | | | | | | | | |
| **Sample types** | | | | | | | **The D group Ma gnesium lignosulfonate (2 g/L)** | | | | | | | | | | | | | | | | **The D group Ma gnesium lignosulfonate (4 g/L)** | | | | | | | | | | | | | | | | | | | **The D group Ma gnesium lignosulfonate (6 g/L)** | | | | | | | | | | | | | | | | **The D group Ma gnesium lignosulfonate (8 g/L)** | | | | | | | | | | **The D group Ma gnesium lignosulfonate (10 g/L)** | | | | | | | | | | | | | | | **The total amount of each component of the sample** | | | | | |
| Concrete | | P.O42.5 +  Ma gnesium lignosulfonate (2 g/L) +  Bacterial additive solution: repeat 12 times | | | | | | | | | | | P.O42.5 +  Ma gnesium lignosulfonate (4 g/L) + Bacterial additive solution: repeat 12 times | | | | | | | | | | | | | | | | | | | | P.O42.5 + Ma gnesium lignosulfonate (6 g/L) +  Bacterial additive solution: repeat 12 times | | | | | | | | | | | | | | | | | P.O42.5+ Ma gnesium lignosulfonate (8 g/L) + Bacterial additive solution: repeat 12 times | | | | | | | | | | | | | | | | P.O42.5 + Ma gnesium lignosulfonate (10 g/L) + Bacterial additive solution: repeat 12 times | | | | | | | | | P.O42.5 used 60 g  Ma gnesium lignosulfonate (2 g/L) solution used 3.84 mL  Ma gnesium lignosulfonate (4 g/L) solution used 3.84 mL  Ma gnesium lignosulfonate (6 g/L) solution used 3.84 mL  Ma gnesium lignosulfonate (8 g/L) solution used 3.84 mL  Ma gnesium lignosulfonate (10 g/L) solution used 3.84 mL  Bacterial additive solution used 18 mL | | | | | | | | | | | | | |
| Yunnan Province Red soil | | | | | | | Red soil in Yunnan Province+Ma gnesium lignosulfonate (2 g/L) +Bacterial additive solution: repeat 3 times | | | | | | | | | | | Red soil in Yunnan Province+Ma gnesium lignosulfonate (4 g/L) +Bacterial additive solution: repeat 3 times | | | | | | | | | | | | | | | | | | | Red soil in Yunnan Province+Ma gnesium lignosulfonate (6 g/L) +Bacterial additive solution: repeat 3 times | | | | | | | | | | | | | | | | | | | Red soil in Yunnan Province+Ma gnesium lignosulfonate (8 g/L) +Bacterial additive solution: repeat 3 times | | | | | | | | | | | Red soil in Yunnan Province+Ma gnesium lignosulfonate (10 g/L) +Bacterial additive solution: repeat 3 times | | | | | | | | | | Red soil used 45 g  Ma gnesium lignosulfonate (2 g/L) solution used 0.9 mL  Ma gnesium lignosulfonate (4 g/L) solution used 0.9 mL  Ma gnesium lignosulfonate (6 g/L) solution used 0.9 mL  Ma gnesium lignosulfonate (8 g/L) solution used 0.9 mL  Ma gnesium lignosulfonate (10 g/L) solution used 0.9 mL  Bacterial additive solution used 4.5 mL | | | | | | | | | | | |
| Henan Province Yellow soil | | | | | | | Yellow soil in Henan Province +Ma gnesium lignosulfonate (2 g/L) +Bacterial additive solution: repeat 3 times | | | | | | | | | | | Yellow soil in Henan Province +Ma gnesium lignosulfonate (4 g/L) +Bacterial additive solution: repeat 3 times | | | | | | | | | | | | | | | | | | | Yellow soil in Henan Province +Ma gnesium lignosulfonate (6 g/L) +Bacterial additive solution: repeat 3 times | | | | | | | | | | | | | | | | | | Yellow soil in Henan Province +Ma gnesium lignosulfonate (8 g/L) +Bacterial additive solution: repeat 3 times | | | | | | | | | | | | Yellow soil in Henan Province +Ma gnesium lignosulfonate (10 g/L) +Bacterial additive solution: repeat 3 times | | | | | | | | | | Yellow soil used 45 g  Ma gnesium lignosulfonate (2 g/L) solution used 0.9 mL  Ma gnesium lignosulfonate (4 g/L) solution used 0.9 mL  Ma gnesium lignosulfonate (6 g/L) solution used 0.9 mL  Ma gnesium lignosulfonate (8 g/L) solution used 0.9 mL  Ma gnesium lignosulfonate (10 g/L) solution used 0.9 mL  Bacterial additive solution used 4.5 mL | | | | | | | | | | | |
| Sichuan Province Purple soil | | | | | | | Purple soil in Sichuan Province+Ma gnesium lignosulfonate (2 g/L) +Bacterial additive solution: repeat 3 times | | | | | | | | | | | Purple soil in Sichuan Province+Ma gnesium lignosulfonate (4 g/L) +Bacterial additive solution: repeat 3 times | | | | | | | | | | | | | | | | | | | Purple soil in Sichuan Province+Ma gnesium lignosulfonate (6 g/L) +Bacterial additive solution: repeat 3 times | | | | | | | | | | | | | | | | | | Purple soil in Sichuan Province+Ma gnesium lignosulfonate (8 g/L) +Bacterial additive solution: repeat 3 times | | | | | | | | | | | Purple soil in Sichuan Province+Ma gnesium lignosulfonate (10 g/L) +Bacterial additive solution: repeat 3 times | | | | | | | | | | | Purple soil used 45 g  Ma gnesium lignosulfonate (2 g/L) solution used 0.9 mL  Ma gnesium lignosulfonate (4 g/L) solution used 0.9 mL  Ma gnesium lignosulfonate (6 g/L) solution used 0.9 mL  Ma gnesium lignosulfonate (8 g/L) solution used 0.9 mL  Ma gnesium lignosulfonate (10 g/L) solution used 0.9 mL  Bacterial additive solution used 4.5 mL | | | | | | | | | | | |
| Jian gsu Province  Cyan Soil | | | | | | | Cyan Soil in Jian gsu Province+Ma gnesium lignosulfonate (2 g/L) +Bacterial additive solution: repeat 3 times | | | | | | | | | | | Cyan Soil in Jian gsu Province+Ma gnesium lignosulfonate (4 g/L) +Bacterial additive solution: repeat 3 times | | | | | | | | | | | | | | | | | | | Cyan Soil in Jian gsu Province+Ma gnesium lignosulfonate (6 g/L) +Bacterial additive solution: repeat 3 times | | | | | | | | | | | | | | | | | | Cyan Soil in Jian gsu Province+Ma gnesium lignosulfonate (8 g/L) +Bacterial additive solution: repeat 3 times | | | | | | | | | | | Cyan Soil in Jian gsu Province+Ma gnesium lignosulfonate (10 g/L) +Bacterial additive solution: repeat 3 times | | | | | | | | | | | Cyan Soil used 45 g  Ma gnesium lignosulfonate (2 g/L) solution used 0.9 mL  Ma gnesium lignosulfonate (4 g/L) solution used 0.9 mL  Ma gnesium lignosulfonate (6 g/L) solution used 0.9 mL  Ma gnesium lignosulfonate (8 g/L) solution used 0.9 mL  Ma gnesium lignosulfonate (10 g/L) solution used 0.9 mL  Bacterial additive solution used 4.5 mL | | | | | | | | | | | |
| Heilon gjian g Province Black soil | | | | | | | Black soil in Heilon gjian g Province+Ma gnesium lignosulfonate (2 g/L) +Bacterial additive solution: repeat 3 times | | | | | | | | | | | Black soil in Heilon gjian g Province+Ma gnesium lignosulfonate (4 g/L) +Bacterial additive solution: repeat 3 times | | | | | | | | | | | | | | | | | | | Black soil in Heilon gjian g Province+Ma gnesium lignosulfonate (6 g/L) +Bacterial additive solution: repeat 3 times | | | | | | | | | | | | | | | | | | Black soil in Heilon gjian g Province+Ma gnesium lignosulfonate (8 g/L) +Bacterial additive solution: repeat 3 times | | | | | | | | | | | Black soil in Heilon gjian g Province+Ma gnesium lignosulfonate (10 g/L) +Bacterial additive solution: repeat 3 times | | | | | | | | | | | Black soil used 45 g  Ma gnesium lignosulfonate (2 g/L) solution used 0.9 mL  Ma gnesium lignosulfonate (4 g/L) solution used 0.9 mL  Ma gnesium lignosulfonate (6 g/L) solution used 0.9 mL  Ma gnesium lignosulfonate (8 g/L) solution used 0.9 mL  Ma gnesium lignosulfonate (10 g/L) solution used 0.9 mL  Bacterial additive solution used 4.5 mL | | | | | | | | | | | |
| **Sample types** | | | | | | | | | **The E group Sodium ligninsulfonate (2 g/L)** | | | | | | | | | | | | | | | | | | | **The E group Sodium ligninsulfonate (4 g/L)** | | | | | | | | | | | | | **The E group Sodium ligninsulfonate (6 g/L)** | | | | | | | | | | | | | | | **The E group Sodium ligninsulfonate (8 g/L)** | | | | | | | | | | **The E group Sodium ligninsulfonate (10 g/L)** | | | | | | | | | | | | **The total amount of each component of the sample** | | | | | | | | | | |
| Concrete | | | | P.O42.5 +Sodium ligninsulfonate (2 g/L) +Bacterial additive solution: repeat 12 times | | | | | | | | | P.O42.5 +Sodium ligninsulfonate (4 g/L) +Bacterial additive solution: repeat 12 times | | | | | | | | | | | | | | | | | | | | P.O42.5 + Sodium ligninsulfonate (6 g/L) +Bacterial additive solution: repeat 12 times | | | | | | | | | | | | | | | | | P.O42.5+ Sodium ligninsulfonate (8 g/L) +Bacterial additive solution: repeat 12 times | | | | | | | | | | | | | | | | P.O42.5 + Sodium ligninsulfonate (10 g/L) +Bacterial additive solution: repeat 12 times | | | | | | | | | P.O42.5 used 60 g  Sodium ligninsulfonate (2 g/L) solution used 3.84 mL  Sodium ligninsulfonate (4 g/L) solution used 3.84 mL  Sodium ligninsulfonate (6 g/L) solution used 3.84 mL  Sodium ligninsulfonate (8 g/L) solution used 3.84 mL  Sodium ligninsulfonate (10 g/L) solution used 3.84 mL  Bacterial additive solution used 18 mL | | | | | | | | | | | | | |
| Yunnan Province Red soil | | | | | Red soil in Yunnan Province+ Sodium ligninsulfonate (2 g/L) +Bacterial additive solution: repeat 3 times | | | | | | | | | | | | | | Red soil in Yunnan Province+ Sodium ligninsulfonate (4 g/L) +Bacterial additive solution: repeat 3 times | | | | | | | | | | | | | | | | | | | Red soil in Yunnan Province+ Sodium ligninsulfonate (6 g/L) +Bacterial additive solution: repeat 3 times | | | | | | | | | | | | | | | | | | Red soil in Yunnan Province+ Sodium ligninsulfonate (8 g/L) +Bacterial additive solution: repeat 3 times | | | | | | | | | | | Red soil in Yunnan Province+ Sodium ligninsulfonate (10 g/L) +Bacterial additive solution: repeat 3 times | | | | | | | | | | Red soil used 45 g  Sodium ligninsulfonate (2 g/L) solution used 0.9 mL  Sodium ligninsulfonate (4 g/L) solution used 0.9 mL  Sodium ligninsulfonate (6 g/L) solution used 0.9 mL  Sodium ligninsulfonate (8 g/L) solution used 0.9 mL  Sodium ligninsulfonate (10 g/L) solution used 0.9 mL  Bacterial additive solution used 4.5 mL | | | | | | | | | | | |
| Henan Province Yellow soil | | | | | Yellow soil in Henan Province + Sodium ligninsulfonate (2 g/L) +Bacterial additive solution: repeat 3 times | | | | | | | | | | | | | | Yellow soil in Henan Province + Sodium ligninsulfonate (4 g/L) +Bacterial additive solution: repeat 3 times | | | | | | | | | | | | | | | | | | | Yellow soil in Henan Province + Sodium ligninsulfonate (6 g/L) +Bacterial additive solution: repeat 3 times | | | | | | | | | | | | | | | | | | Yellow soil in Henan Province + Sodium ligninsulfonate (8 g/L) +Bacterial additive solution: repeat 3 times | | | | | | | | | | | Yellow soil in Henan Province + Sodium ligninsulfonate (10 g/L) +Bacterial additive solution: repeat 3 times | | | | | | | | | | Yellow soil used 45 g  Sodium ligninsulfonate (2 g/L) solution used 0.9 mL  Sodium ligninsulfonate (4 g/L) solution used 0.9 mL  Sodium ligninsulfonate (6 g/L) solution used 0.9 mL  Sodium ligninsulfonate (8 g/L) solution used 0.9 mL  Sodium ligninsulfonate (10 g/L) solution used 0.9 mL  Bacterial additive solution used 4.5 mL | | | | | | | | | | | |
| Sichuan Province Purple soil | | | | | Purple soil in Sichuan Province+ Sodium ligninsulfonate (2 g/L) +Bacterial additive solution: repeat 3 times | | | | | | | | | | | | | | | Purple soil in Sichuan Province+ Sodium ligninsulfonate (4 g/L) +Bacterial additive solution: repeat 3 times | | | | | | | | | | | | | | | | | | Purple soil in Sichuan Province+ Sodium ligninsulfonate (6 g/L) +Bacterial additive solution: repeat 3 times | | | | | | | | | | | | | | | | | | | Purple soil in Sichuan Province+ Sodium ligninsulfonate (8 g/L) +Bacterial additive solution: repeat 3 times | | | | | | | | | Purple soil in Sichuan Province+ Sodium ligninsulfonate (10 g/L) +Bacterial additive solution: repeat 3 times | | | | | | | | | | | | | | Purple soil used 45 g  Sodium ligninsulfonate (2 g/L) solution used 0.9 mL  Sodium ligninsulfonate (4 g/L) solution used 0.9 mL  Sodium ligninsulfonate (6 g/L) solution used 0.9 mL  Sodium ligninsulfonate (8 g/L) solution used 0.9 mL  Sodium ligninsulfonate (10 g/L) solution used 0.9 mL  Bacterial additive solution used 4.5 mL | | | | | | | | |
| Jian gsu Province  Cyan Soil | | | | | Cyan Soil in Jian gsu Province+ Sodium ligninsulfonate (2 g/L) +Bacterial additive solution: repeat 3 times | | | | | | | | | | | | | | | Cyan Soil in Jian gsu Province+ Sodium ligninsulfonate (4 g/L) +Bacterial additive solution: repeat 3 times | | | | | | | | | | | | | | | | | | Cyan Soil in Jian gsu Province+ Sodium ligninsulfonate (6 g/L) +Bacterial additive solution: repeat 3 times | | | | | | | | | | | | | | | | | | | Cyan Soil in Jian gsu Province+ Sodium ligninsulfonate (8 g/L) +Bacterial additive solution: repeat 3 times | | | | | | | | | Cyan Soil in Jian gsu Province+ Sodium ligninsulfonate (10 g/L) +Bacterial additive solution: repeat 3 times | | | | | | | | | | | Cyan Soil used 45 g  Sodium ligninsulfonate (2 g/L) solution used 0.9 mL  Sodium ligninsulfonate (4 g/L) solution used 0.9 mL  Sodium ligninsulfonate (6 g/L) solution used 0.9 mL  Sodium ligninsulfonate (8 g/L) solution used 0.9 mL  Sodium ligninsulfonate (10 g/L) solution used 0.9 mL  Bacterial additive solution used 4.5 mL | | | | | | | | | | | |
| Heilon gjian g Province Black soil | | | | | | Black soil in Heilon gjian g Province+ Sodium ligninsulfonate (2 g/L) +Bacterial additive solution: repeat 3 times | | | | | | | | | | | | | | | Black soil in Heilon gjian g Province+ Sodium ligninsulfonate (4 g/L) +Bacterial additive solution: repeat 3 times | | | | | | | | | | | | | | | | | | Black soil in Heilon gjian g Province+ Sodium ligninsulfonate (6 g/L) +Bacterial additive solution: repeat 3 times | | | | | | | | | | | | | | | | | | Black soil in Heilon gjian g Province+ Sodium ligninsulfonate (8 g/L) +Bacterial additive solution: repeat 3 times | | | | | | | | | Black soil in Heilon gjian g Province+ Sodium ligninsulfonate (10 g/L) +Bacterial additive solution: repeat 3 times | | | | | | | | | | | Black soil used 45 g  Sodium ligninsulfonate (2 g/L) solution used 0.9 mL  Sodium ligninsulfonate (4 g/L) solution used 0.9 mL  Sodium ligninsulfonate (6 g/L) solution used 0.9 mL  Sodium ligninsulfonate (8 g/L) solution used 0.9 mL  Sodium ligninsulfonate (10 g/L) solution used 0.9 mL  Bacterial additive solution used 4.5 mL | | | | | | | | | | | |
| **Sample types** | | | | | | | **The F group Riboflavin (2 g/L)** | | | | | | | | | | | | | | | **The F group Riboflavin (4 g/L)** | | | | | | | | | | | | | | | | | | **The F group Riboflavin (6 g/L)** | | | | | | | | | | | | | | | | | | **The F group Riboflavin (8 g/L)** | | | | | | | | | | | | **The F group Riboflavin**  **(10 g/L)** | | | | | | | | | | | | | | **The total amount of each component of the sample** | | | | |
| Concrete | P.O42.5 +Riboflavin (2 g/L) +Bacterial additive solution: repeat 12 times | | | | | | | | | | | | | P.O42.5 +Riboflavin (4 g/L) +Bacterial additive solution: repeat 12 times | | | | | | | | | | | | | | | | | | | | P.O42.5 + Riboflavin (6 g/L) +Bacterial additive solution: repeat 12 times | | | | | | | | | | | | | | | | | | P.O42.5+ Riboflavin (8 g/L) +Bacterial additive solution: repeat 12 times | | | | | | | | | | | | | | P.O42.5 + Riboflavin (10 g/L) +Bacterial additive solution: repeat 12 times | | | | | | | | | | P.O42.5 used 60 g  Riboflavin (2 g/L) solution used 3.84 mL  Riboflavin (4 g/L) solution used 3.84 mL  Riboflavin (6 g/L) solution used 3.84 mL  Riboflavin (8 g/L) solution used 3.84 mL  Riboflavin (10 g/L) solution used 3.84 mL  Bacterial additive solution used 18 mL | | | | | | | | | | | | |
| Yunnan Province  Red soil | | | | | | | | Red soil in Yunnan Province+ Riboflavin (2 g/L) +Bacterial additive solution: repeat 3 times | | | | | | | | | | | | | | | | | Red soil in Yunnan Province+ Riboflavin (4 g/L) +Bacterial additive solution: repeat 3 times | | | | | | | | | | | | | | | | | | | Red soil in Yunnan Province+ Riboflavin (6 g/L) +Bacterial additive solution: repeat 3 times | | | | | | | | | | | | | | | | Red soil in Yunnan Province+ Riboflavin (8 g/L) +Bacterial additive solution: repeat 3 times | | | | | | | | | | Red soil in Yunnan Province+ Riboflavin (10 g/L) +Bacterial additive solution: repeat 3 times | | | | | | | Red soil used45 g  Riboflavin (2 g/L) solution used 0.9 mL  Riboflavin (4 g/L) solution used 0.9 mL  Riboflavin (6 g/L) solution used 0.9 mL  Riboflavin (8 g/L) solution used 0.9 mL  Riboflavin (10 g/L) solution used 0.9 mL  Bacterial additive solution used 4.5 mL | | | | | | | | | | | |
| Henan Province Yellow soil | | | | | | | | Yellow soil in Henan Province + Riboflavin (2 g/L) +Bacterial additive solution: repeat 3 times | | | | | | | | | | | | | | | | Yellow soil in Henan Province + Riboflavin (4 g/L) +Bacterial additive solution: repeat 3 times | | | | | | | | | | | | | | | | | | | Yellow soil in Henan Province + Riboflavin (6 g/L) +Bacterial additive solution: repeat 3 times | | | | | | | | | | | | | | | | Yellow soil in Henan Province + Riboflavin (8 g/L) +Bacterial additive solution: repeat 3 times | | | | | | | | | | Yellow soil in Henan Province + Riboflavin (10 g/L) +Bacterial additive solution: repeat 3 times | | | | | | | | Yellow soil used 45 g  Riboflavin (2 g/L) solution used 0.9 mL  Riboflavin (4 g/L) solution used 0.9 mL  Riboflavin (6 g/L) solution used 0.9 mL  Riboflavin (8 g/L) solution used 0.9 mL  Riboflavin (10 g/L) solution used 0.9 mL  Bacterial additive solution used 4.5 mL | | | | | | | | | | | |
| Sichuan Province Purple soil | | | | | | | | Purple soil in Sichuan Province+ Riboflavin (2 g/L) +Bacterial additive solution: repeat 3 times | | | | | | | | | | | | | | | | Purple soil in Sichuan Province+ Riboflavin (4 g/L) +Bacterial additive solution: repeat 3 times | | | | | | | | | | | | | | | | | | | Purple soil in Sichuan Province+ Riboflavin (6 g/L) +Bacterial additive solution: repeat 3 times | | | | | | | | | | | | | | | | Purple soil in Sichuan Province+ Riboflavin (8 g/L) +Bacterial additive solution: repeat 3 times | | | | | | | Purple soil in Sichuan Province+ Riboflavin (10 g/L) +Bacterial additive solution: repeat 3 times | | | | | | | | | | | Purple soil used 45 g  Riboflavin (2 g/L) solution used 0.9 mL  Riboflavin (4 g/L) solution used 0.9 mL  Riboflavin (6 g/L) solution used 0.9 mL  Riboflavin (8 g/L) solution used 0.9 mL  Riboflavin (10 g/L) solution used 0.9 mL  Bacterial additive solution used 4.5 mL | | | | | | | | | | | |
| Jian gsu Province  Cyan Soil | | | | | | | | Cyan Soil in Jian gsu Province+ Riboflavin (2 g/L) +Bacterial additive solution: repeat 3 times | | | | | | | | | | | | | | | | Cyan Soil in Jian gsu Province+ Riboflavin (4 g/L) +Bacterial additive solution: repeat 3 times | | | | | | | | | | | | | | | | | | | Cyan Soil in Jian gsu Province+ Riboflavin (6 g/L) +Bacterial additive solution: repeat 3 times | | | | | | | | | | | | | | | | Cyan Soil in Jian gsu Province+ Riboflavin (8 g/L) +Bacterial additive solution: repeat 3 times | | | | | | | Cyan Soil in Jian gsu Province+ Riboflavin (10 g/L) +Bacterial additive solution: repeat 3 times | | | | | | | | | | | Cyan Soil used 45 g  Riboflavin (2 g/L) solution used 0.9 mL  Riboflavin (4 g/L) solution used 0.9 mL  Riboflavin (6 g/L) solution used 0.9 mL  Riboflavin (8 g/L) solution used 0.9 mL  Riboflavin (10 g/L) solution used 0.9 mL  Bacterial additive solution used 4.5 mL | | | | | | | | | | | |
| Heilon gjian g Province Black soil | | | | | | | | Black soil in Heilon gjian g Province+ Riboflavin (2 g/L) +Bacterial additive solution: repeat 3 times | | | | | | | | | | | | | | | | | | Black soil in Heilon gjian g Province+ Riboflavin (4 g/L) +Bacterial additive solution: repeat 3 times | | | | | | | | | | | | | | | | | | | Black soil in Heilon gjian g Province+ Riboflavin (6 g/L) +Bacterial additive solution: repeat 3 times | | | | | | | | | | | | | | | | Black soil in Heilon gjian g Province+ Riboflavin (8 g/L) +Bacterial additive solution: repeat 3 times | | | | | | | | | | Black soil in Heilon gjian g Province+ Riboflavin (10 g/L) +Bacterial additive solution: repeat 3 times | | | | | | | | | | | | | | Black soil used 45 g  Riboflavin (2 g/L) solution used 0.9 mL  Riboflavin (4 g/L) solution used 0.9 mL  Riboflavin (6 g/L) solution used 0.9 mL  Riboflavin (8 g/L) solution used 0.9 mL  Riboflavin (10 g/L) solution used 0.9 mL  Bacterial additive solution used 4.5 mL | | | |
| **Sample types** | | | | | | | | **The G group CaCl_2_ (0.42 g/L)** | | | | | | | | | | | | | | | | | | | **The G group CaCl_2_ (0.84 g/L)** | | | | | | | | | | | | | | | | | | | **The G group CaCl_2_ (1.26 g/L)** | | | | | | | | | | | | | | | | **The G group CaCl_2_ (1.68 g/L)** | | | | | | | | | | **The G group CaCl_2_ (2.10 g/L)** | | | | | | | | | | | | | | **The total amount of each component of the sample** | | |
| Concrete | | | P.O42.5 + CaCl_2_ (0.42 g/L) +Bacterial additive solution: repeat 12 times | | | | | | | | | | | | P.O42.5 + CaCl_2_ (0.84 g/L) +Bacterial additive solution: repeat 12 times | | | | | | | | | | | | | | | | | | | | P.O42.5 + CaCl_2_ (1.26 g/L) +Bacterial additive solution: repeat 12 times | | | | | | | | | | | | | | | | | | P.O42.5+ CaCl_2_ (1.68 g/L) +Bacterial additive solution: repeat 12 times | | | | | | | | | | | | | P.O42.5 +CaCl_2_ (2.10 g/L) +Bacterial additive solution: repeat 12 times | | | | | | | | | | | | | | | | P.O42.5 used 60 g  CaCl_2_ (0.42 g/L) solution used 3.84 mL  CaCl_2_ (0.84 g/L) solution used 3.84 mL  CaCl_2_ (1.26 g/L) solution used 3.84 mL  CaCl_2_ (1.68 g/L) solution used 3.84 mL  CaCl_2_(2.10 g/L) solution used 3.84 mL  Bacterial additive solution used 18 mL | | | | | | |

Table S2: Preparation formula of concrete (P.O42.5) samples for Raman test.

| **Grouping of additives and serial number of samples** | | | | | | |
| --- | --- | --- | --- | --- | --- | --- |
| **Sample types** | **The A group** | | | | | **The total amount of each component of the sample** |
| Concrete | P.O42.5+H_2_O | | | | | P.O42.5 used 100.06 g  H_2_O used 25 mL |
| **Sample types** | **The B group** | | | | | **The total amount of each component of the sample** |
| Concrete | P.O42.5+H_2_O+ Bacterial additive solution | | | | | P.O42.5 used 100.08 g  H_2_O used 25 mL  Bacterial additive solution used 3.5 mL |
| **Sample types** | **The C group** Calcium lignosulphonate (2 g/L） | **The C group** Calcium lignosulphonate (4 g/L） | **The C group** Calcium lignosulphonate (6 g/L） | **The C group** Calcium lignosulphonate (8 g/L） | **The C group** Calcium lignosulphonate (10 g/L） | **The total amount of each component of the sample** |
| Concrete | P.O42.5 +Calcium lignosulphonate (2 g/L）+ Bacterial additive solution | P.O42.5 +Calcium lignosulphonate (4 g/L）+ Bacterial additive solution | P.O42.5 + Calcium lignosulphonate (6 g/L）+ Bacterial additive solution | P.O42.5+ Calcium lignosulphonate (8 g/L）+ Bacterial additive solution | P.O42.5 + Calcium lignosulphonate (10 g/L）+ Bacterial additive solution | P.O42.5 used 500.53 g  Calcium lignosulphonate (2 g/L）solution used 25 mL  Calcium lignosulphonate (4 g/L）solution used 25 mL  Calcium lignosulphonate (6 g/L）solution used 25 mL  Calcium lignosulphonate (8 g/L）solution used 25 mL  Calcium lignosulphonate (10 g/L）solution used 25 mL  Bacterial additive solution used 18 mL |

Table S3: The proportion of each element in EDS detection.

| **Spectrum label** | **Atlas 1** | **Atlas 2** | **Atlas 3** |
| --- | --- | --- | --- |
| C | 17.48 | 15.93 | 16.17 |
| O | 38.01 | 38.51 | 38.20 |
| Na | 0.90 | 0.79 | 0.74 |
| M g | 0.37 | 0.35 | 0.28 |
| Al | 0.45 | 0.38 | 0.54 |
| Si | 2.66 | 1.87 | 3.18 |
| P | 2.60 | 2.05 | 2.23 |
| S | 1.19 | 1.18 | 1.18 |
| Cl | 1.45 | 1.57 | 1.62 |
| K | 1.15 | 0.87 | 0.94 |
| Ca | 33.74 | 36.50 | 34.92 |
| Total | 100.00 | 100.00 | 100.00 |

Table S4: Comparison between traditional MICP process and our MICP process.

|  | **Traditional MICP** | **Our MICP** |
| --- | --- | --- |
| **Bacteria** | Pathogenic bacteria, such as Bacillus cereus ,  Streptococcus pasteurii , Salmonella pasteurii ,  drinking water-borne pathogens, Pseudomonas  aeruginosa, Bacillus sphaericus, Bacillus cohnii,  and detoxifying bacteria. | Non-pathogenic Bacteria, Bacillus subtilis 168. |
| **Energy source** |  |  |
| **to drive the** | Urea | Calcium lignosulfonate |
| **MICP process** |  |  |
| **Impact** | The presence of by-product ammonia, excessive  ammonia will accelerate eutrophication, and  depletion of dissolved oxygen in the environment . | The process entirely prevents the production of ammonia, increases calcite yield, and  minimizes the environmental impact. |
